# Dysfunctional tear syndrome: dry eye disease and associated tear film disorders – new strategies for diagnosis and treatment

**DOI:** 10.1097/01.icu.0000512373.81749.b7

**Published:** 2017-01-16

**Authors:** Mark S. Milner, Kenneth A. Beckman, Jodi I. Luchs, Quentin B. Allen, Richard M. Awdeh, John Berdahl, Thomas S. Boland, Carlos Buznego, Joseph P. Gira, Damien F. Goldberg, David Goldman, Raj K. Goyal, Mitchell A. Jackson, James Katz, Terry Kim, Parag A. Majmudar, Ranjan P. Malhotra, Marguerite B. McDonald, Rajesh K. Rajpal, Tal Raviv, Sheri Rowen, Neda Shamie, Jonathan D. Solomon, Karl Stonecipher, Shachar Tauber, William Trattler, Keith A. Walter, George O. Waring, Robert J. Weinstock, William F. Wiley, Elizabeth Yeu

**Affiliations:** a Yale University School of Medicine, New Haven; b The Eye Center of Southern Connecticut, Hamden, Connecticut; c Ohio State University, Columbus; d Comprehensive Eye Care of Central Ohio, Westerville, Ohio; e Hofstra Northwell School of Medicine, Hempstead; f South Shore Eye Care, Wantagh, New York; g Florida Vision Institute, Jupiter; h Bascom Palmer Eye Institute, Florida International University, and Center for Excellence in Eye Care, Miami, Florida; i Vance Thompson Vision, Sioux Falls, South Dakota; j Northeastern Eye Institute, Scranton; k Commonwealth Medical College, Scranton, Pennsylvania; l Ophthalmology Consultants, St. Louis, Missouri; m Jules Stein Eye Institute, Los Angeles; n Wolstan & Goldberg Eye Associates, Torrance, California; o Palm Beach Gardens, Florida; p Rush University Medical Center, Chicago; q Chicago Eye Specialists; r University of Chicago Hospitals, Chicago; s Jacksoneye, Lake Villa; t Midwest Center for Sight, Des Plaines, Illinois; u Duke Eye Center, Durham, North Carolina; v Chicago Cornea Consultants, Ltd, Hoffman Estates, Illinois; w Washington University Department of Ophthalmology and Ophthalmology Associates, St. Louis, Missouri; x NYU Langone Medical Center, New York, New York; y Tulane University School of Medicine, New Orleans, Louisiana; z Ophthalmic Consultants of Long Island, Lynbrook, New York; aa The Center for Ocular Surface Excellence of New Jersey, Woodland Park, New Jersey; bb Georgetown University Medical Center, George Washington University Medical Center, Washington, DC; cc New York Eye and Ear Infirmary of Mount Sinai and Eye Center of New York, New York, New York; dd NVision EyeCenters of Newport Beach, Newport Beach, California; ee University of Maryland, Baltimore, Maryland; ff Advanced Vision Care, Century City, California; gg Keck School of Medicine, University of Southern California, Los Angeles; hh Bowie Vision Institute, Bowie, Maryland; ii University of North Carolina and TLC Laser Eye Centers, Greensboro, North Carolina; jj Mercy Eye Specialists, Springfield, Missouri; kk Wake Forest University, Winston-Salem, North Carolina; ll Storm Eye Institute and Magill Vision Center, Medical University of South Carolina, Charleston; mm Clemson University, Mt. Pleasant, South Carolina; nn University of South Florida, Tampa; oo The Eye Institute of West Florida, Largo, Florida; pp Cleveland Eye Clinic, Clear Choice Custom LASIK Center, Brecksville, Ohio; qq Eastern Virginia Medical School and Virginia Eye Consultants, Norfolk, Virginia, USA; ∗ Mark S. Milner, Kenneth A. Beckman, and Jodi I. Luchs are co-chairs.

## Abstract

Dysfunctional tear syndrome (DTS) is a common and complex condition affecting the ocular surface. The health and normal functioning of the ocular surface is dependent on a stable and sufficient tear film. Clinician awareness of conditions affecting the ocular surface has increased in recent years because of expanded research and the publication of diagnosis and treatment guidelines pertaining to disorders resulting in DTS, including the Delphi panel treatment recommendations for DTS (2006), the International Dry Eye Workshop (DEWS) (2007), the Meibomian Gland Dysfunction (MGD) Workshop (2011), and the updated Preferred Practice Pattern guidelines from the American Academy of Ophthalmology pertaining to dry eye and blepharitis (2013). Since the publication of the existing guidelines, new diagnostic techniques and treatment options that provide an opportunity for better management of patients have become available. Clinicians are now able to access a wealth of information that can help them obtain a differential diagnosis and treatment approach for patients presenting with DTS. This review provides a practical and directed approach to the diagnosis and treatment of patients with DTS, emphasizing treatment that is tailored to the specific disease subtype as well as the severity of the condition.

## INTRODUCTION

**FIGURE 1:**
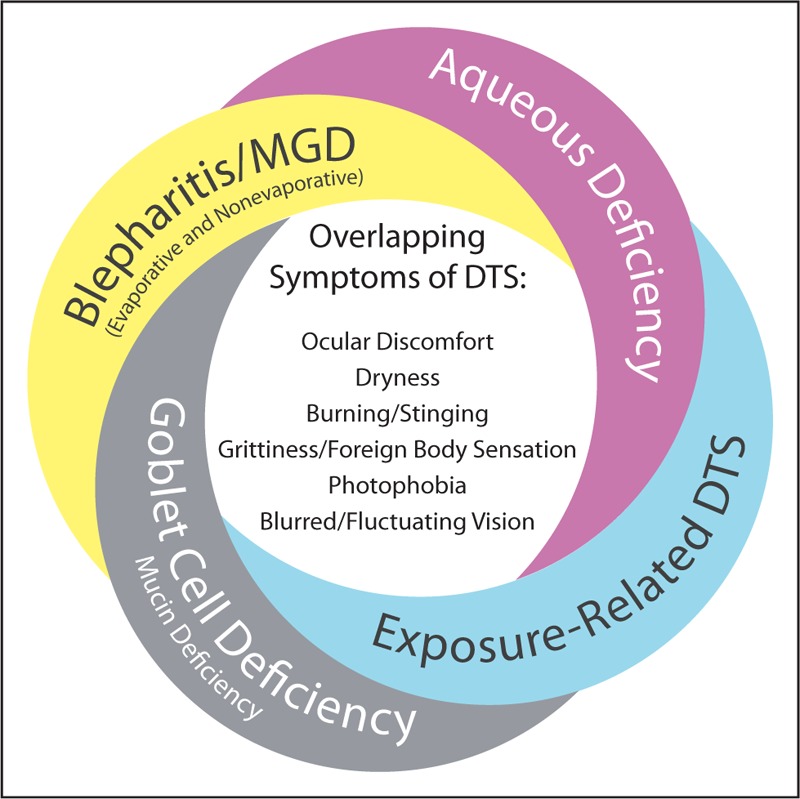
Overlap of symptoms in dysfunctional tear syndrome (DTS) subtypes. There is a substantial overlap in the patient-reported symptoms of the types of DTS. Patients with subtypes of aqueous deficiency, blepharitis/meibomian gland dysfunction (MGD) – evaporative and nonevaporative – goblet cell deficiency/mucin deficiency, and exposure-related forms of DTS, including exposure keratopathy, may report symptoms of ocular discomfort, dryness, burning/stinging, grittiness or a foreign body sensation, photophobia, and blurred/fluctuating vision. Clinical evaluation with a battery of assessments and diagnostic techniques are needed for a differential diagnosis.

Management of dysfunctional tear syndrome [1] (DTS) is often a source of frustration for eye care professionals and patients. Several factors create challenges in the diagnosis and management of DTS. These include the lack of correlation between signs and symptoms [2,3], overlap among symptoms of different DTS subtypes (Fig. 1) [2], complex etiology, poorly understood pathophysiology of DTS-associated conditions, historically limited range of diagnostic tests, limited number of US Food and Drug Administration-approved treatment options, and the potential progressive nature of the condition. Poor patient compliance with follow-up visits also contributes significantly to the difficulties encountered when treating patients with DTS.

Clinician awareness of conditions affecting the ocular surface has increased in recent years because of new clinical research and the publication of diagnostic and treatment guidelines for disorders resulting in DTS. These guidelines include the Delphi panel treatment recommendations for DTS (2006) [1], the International Dry Eye WorkShop (DEWS) (2007) [4], the International Workshop on Meibomian Gland Dysfunction (MGD) (2011) [5], and the updated Preferred Practice Pattern guidelines from the American Academy of Ophthalmology pertaining to dry eye and blepharitis (2013) [6,7]. These guidelines generally recommend treatment based on the severity of the condition for the subtypes of DTS. New diagnostic methods and pharmacologic treatments that can be used to further inform severity-based decisions to help better manage DTS and associated masquerading conditions have since become available.

To combine the latest evidence-based approaches for diagnosis and management of DTS with existing guideline-based approaches, we convened a specialty panel with experts from the Cornea, External Disease, and Refractive Society (CEDARS), hereafter referred to as the DTS Panel, to provide a clinical approach to using the latest diagnostic tools and guidelines to direct treatment that is tailored to the specific disease subtype(s). The evidence for a comprehensive range of diagnostic methods, including recently developed techniques, and a comprehensive review of established and new treatment modalities for managing DTS and its subtypes is reviewed herein. Case studies are included to demonstrate how the new approaches can be applied to specific clinical scenarios.

## DIAGNOSIS-BASED INDIVIDUALIZED TREATMENT APPROACH

The primary goal of the DTS Panel was to provide an approach for improved outcomes in the treatment of patients with DTS through differential diagnosis and directed treatment. Toward this end, we started by defining DTS as a disorder of the tear film in quality and/or quantity, which is caused by a range of etiologies and involves abnormalities in one or more components of the tear film, resulting in a constellation of signs and symptoms affecting the ocular surface. Any alteration in the quantity and/or quality of the tear film can result in DTS, a chronic condition with multiple subtypes that include dry eye disease (DED) and associated tear film disorders. It has been shown that without proper diagnosis and management, DTS can result in profound degradation in quality of life and visual function-based activities (i.e., reading, driving, and computer use), the extent of which correlates with the severity of the condition [8]. Additionally, DTS can negatively affect surgical outcomes, such as those of cataract and refractive surgical procedures [9,10]. The overlap and frequent comorbidity of DED and other conditions affecting the quantity or quality of the tear film require careful examination of patients with DTS. Achieving a differential diagnosis and classification into specific disease-state subtypes allows a directed, individualized treatment approach.

**FIGURE 2:**
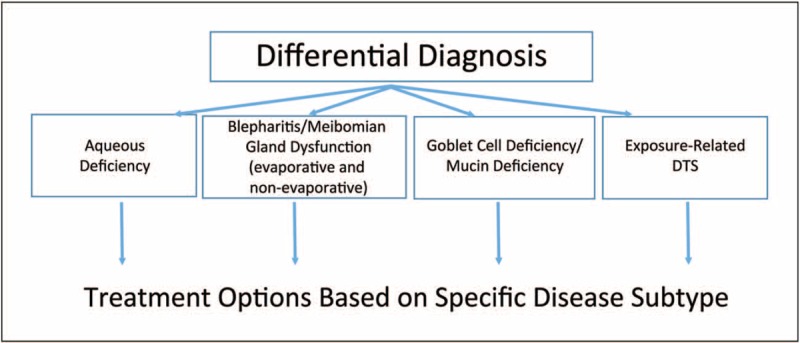
Flow chart of the DTS Panel clinical approach. A differential diagnosis is achieved through the DTS Panel clinical approach through examination of patients via a series of clinical assessments and diagnostic techniques. Patients with dysfunctional tear syndrome (DTS) are classified to one or more of the four DTS subtypes: aqueous deficiency, blepharitis/meibomian gland dysfunction, goblet cell deficiency/mucin deficiency, and exposure-related DTS. Treatment options are determined on the basis of the specific DTS subtype(s) identified, thus allowing for a directed treatment approach.

Four main subtype classifications used by the DTS Panel approach are as follows: first, aqueous deficiency; second, blepharitis/MGD (evaporative and nonevaporative); third, goblet cell deficiency/mucin deficiency; and fourth, exposure-related DTS (Fig. 2). Following the clinical assessment and evaluation of a patient with DTS, each patient can be classified into one or more of the disease-state subtypes (the presence of multiple subtypes within a patient is common). Identifying and treating other conditions or ‘DTS co-conspirators’ that may masquerade as or contribute to the signs and symptoms of DTS are equally important.

This approach can be used with new and current patients reporting DTS symptoms (e.g., ocular dryness, discomfort, burning/stinging, and grittiness/foreign body sensation), patients reporting a fluctuation in vision or other nonspecific visual obscurations, referred patients with a history of dry eye or blepharitis, and patients who are being evaluated for laser vision correction or cataract surgery, particularly those who may use multifocal or other premium intraocular lenses.

Published information supporting the use of new diagnostic tests, recently approved therapies, and the potential role of novel or investigational therapies for patients with DTS was evaluated on a tiered basis, ranging from level I through level III, depending on the rigor of the study design, with level I denoting evidence from a randomized controlled clinical trial (or equivalent), level II indicating evidence from a controlled study that was not randomized or involved a cohort-based study at one or more centers, and level III indicating case studies, published meeting abstracts, or expert opinions. The DTS Panel compiled the published literature (i.e., reports of clinical trials, case reports, and meeting presentations) and presented the information for a review discussion at panel working meetings, which used voting to achieve a consensus. The studies cited were deemed the most relevant to the individual topic.

### Step 1: patient history

Table 1 summarizes the types of information that should be collected from a patient with potential DTS. Recording a thorough medical and ocular history, including surgical history, provides the initial context for evaluating the patient. Current and past use of systemic and ocular medications is also important to capture at this stage. Past or current therapy for conditions such as DED, blepharitis, and any allergies, even treatments that were regarded as ‘failures’, should be noted and queried. The timing, frequency, and severity of the chief complaints or symptoms reported by the patient also offer initial insight into the classification of the DTS subtype(s).

**Table 1:**
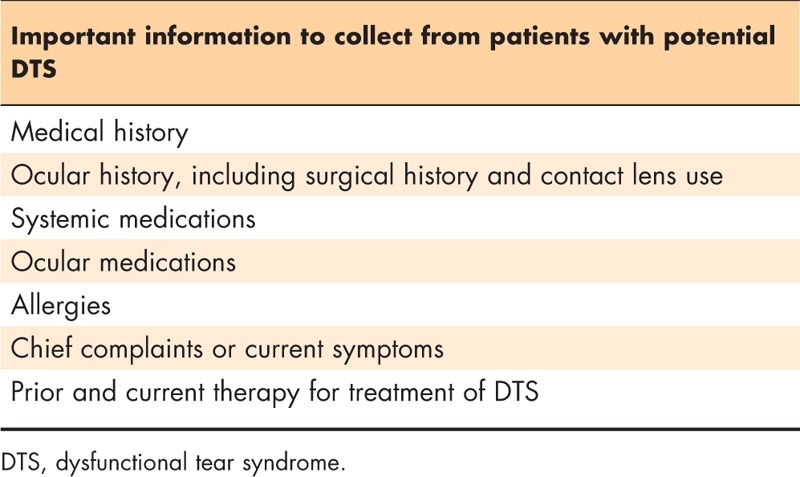
Patient history

### Step 2: preliminary assessments

A technician or other member of the office staff may perform the initial assessment of a patient with potential DTS; however, in some practices, the clinician may conduct this part of the evaluation. Table 2 lists the preliminary assessments recommended by the authors at this point. Consistency in the conduct of each of the assessments among staff members is critical because the results of the diagnostic tests at the initial visit will serve as the baseline for comparison with follow-up visits. Patients with DTS often report blurred vision or intermittent distortion of vision [11]. As such, evaluation of the patient's visual acuity, with and without his or her current corrective glasses or lenses, and an assessment of any refraction necessary to achieve best-corrected visual acuity is an important portion of the preliminary assessment. In addition to assessment of visual acuity, patients should be queried regarding the nature and frequency of any reports of fluctuation in vision. Once the visual acuity and refraction assessments are complete, the patient may be assessed using a questionnaire.

**Table 2:**
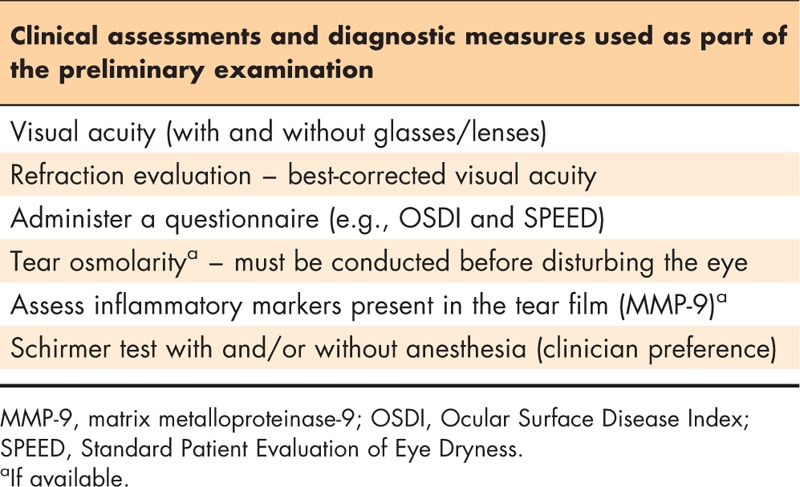
Preliminary assessments performed by the technician or clinician

Prior to conducting any additional assessments involving contact with the ocular surface, tear film osmolarity can be evaluated if the necessary equipment is available [12–15]. Following this evaluation, the relative level of ocular inflammation (common among all subtypes of DTS), as measured by quantifying the level of inflammatory mediator markers, such as matrix metalloproteinase (MMP)-9, in the tear film can be assessed if the laboratory equipment or diagnostic device is available [16–18]. Finally, the patient's relative tear volume and secretion should be measured via a Schirmer strip test [15]. Depending on the patient's response to contact with the ocular surface as part of the preliminary assessments, some of the assessments may need to be conducted at a follow-up visit if significant reflex tearing is observed. The ‘Diagnostics and clinical assessments’ section presents additional detailed information pertaining to the description and interpretation of the diagnostic tests described previously.

### Step 3: primary assessments

The physician or lead clinician generally conducts the primary assessments and tests used in the diagnosis of DTS. Table 3 presents a summary description of the following primary assessments and diagnostic procedures that are recommended at this stage:

(1) Conduct a thorough review of the patient's medical, ocular, and surgical history as well as the nature and frequency of past and current medication use, including any changes that have been noted since a prior visit.
(2) Discuss the patient's responses to the questionnaire or other instrument to help clarify the relative frequency and timing (diurnal pattern) of DTS-related symptoms and any effect on daily activities, such as reading, driving, or computer use. During the review of the patient's medical information and discussion of questionnaire responses, behaviors that may contribute to ocular irritation, such as mucus fishing, may be observed. The responses to the questionnaire may provide insight regarding the severity level of the DTS as well.
(3) Conduct an external examination of the patient's skin, adnexa, and eyelids to provide additional insight toward a differential diagnosis. Express the meibomian glands for each eyelid. Emphasis on the eyelid margin, lashes, and lid closure is important to note any anatomic defects, malposition of the tissue, partial or incomplete blinking, debris, and alteration of the meibum or inflammation of the orifices of the meibomian glands (Fig. 2).
(4) Conduct a slit-lamp examination to detect abnormalities that are essential in determining a differential diagnosis of DTS. The eyelids, including features of the anterior and posterior lid margin, and lashes, should be examined carefully. Additionally, the upper lids should be everted to allow direct visualization of the tarsal conjunctiva. Observe structures of the anterior segment, noting the relative severity of any discernible inflammation or other abnormalities associated with ocular structures. The relative height of the inferior tear meniscus should also be evaluated at this step of the examination. Practitioners may note a normal or decreased tear meniscus during a standard examination; a tear meniscus height of less than 0.3 mm is considered abnormal [15]; however, in the experience of the authors, clinicians may often estimate the tear meniscus and make a subjective determination about whether it is decreased.
(5) Instill fluorescein dye (2–5 μl of 1 or 2% sodium fluorescein or a commercially available fluorescein preparation) via micropipette, a device designed to precisely deliver a small amount of liquid, or through the application of physiological saline-moistened fluorescein strips to the ocular surface for visualization of the tear film and any damage to the corneal epithelium when viewed through the slit-lamp with cobalt blue light and the addition of a Wratten (yellow) filter, if available. Waiting approximately 2 min following the application of the dye to the ocular surface may enhance the visible staining pattern [6]. In general clinical practice, specialty devices, such as a micropipette and slit-lamp filter, may not be available. Ocular surface staining may still be observed through the application of dye via a fluorescein strip or a commercially available fluorescein preparation and direct illumination with the slit-lamp. The severity and location/pattern of corneal fluorescein staining should be carefully noted.
(6) Assess the tear break-up time (TBUT) during this part of the examination by measuring the time between the patient's last blink and the appearance of the first patch of discontinuity in the tear film. Replicating the test two to three times per eye will provide a more reliable result to determine whether the patient has a rapid TBUT (<10 s) [6,19]. Application of vital stains, including lissamine green and/or rose bengal, allows for a similar level of observation of the integrity of the conjunctiva and cornea [20,21]. The ‘Diagnostics and clinical assessments’ section presents additional detailed information pertaining to the description and interpretation of the diagnostic tests described previously.

**Table 3:**
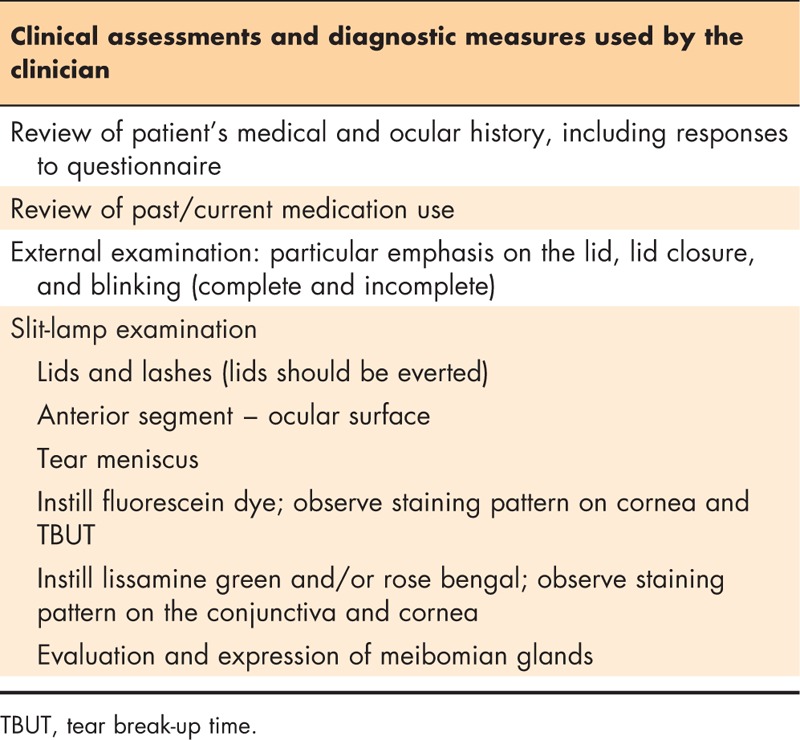
Primary assessments and diagnostic procedures performed by the clinician

### Step 4: additional diagnostic tests

Based on the patient history, clinical findings, and results of the previous diagnostic procedures, additional testing may be necessary or useful in diagnosing DTS. Table 4 briefly describes additional tests that may be used. Although specialized equipment may be needed to conduct certain procedures, other tests may need only basic materials. Corneal topography (keratography) may be included as a diagnostic test in a DTS evaluation, particularly if a patient reports distortion of vision. Optical coherence tomography (OCT) may be used to provide a quantitative assessment of the tear meniscus. Double-pass wave front assessment may be used to provide a functional analysis of the optical characteristics of the tear film based on the light scatter. Interferometry and meibography are other tests that may be conducted, as necessary, if the equipment is available to provide information pertaining to the outer lipid component of the tear film and the relative anatomy of the meibomian glands present within the lids. A simple evaluation of a patient's corneal sensation (normal, decreased, or none) may be determined through the use of a cotton swab, tip of a tissue, nonwaxed dental floss, or a similar substitute.

**Table 4:**
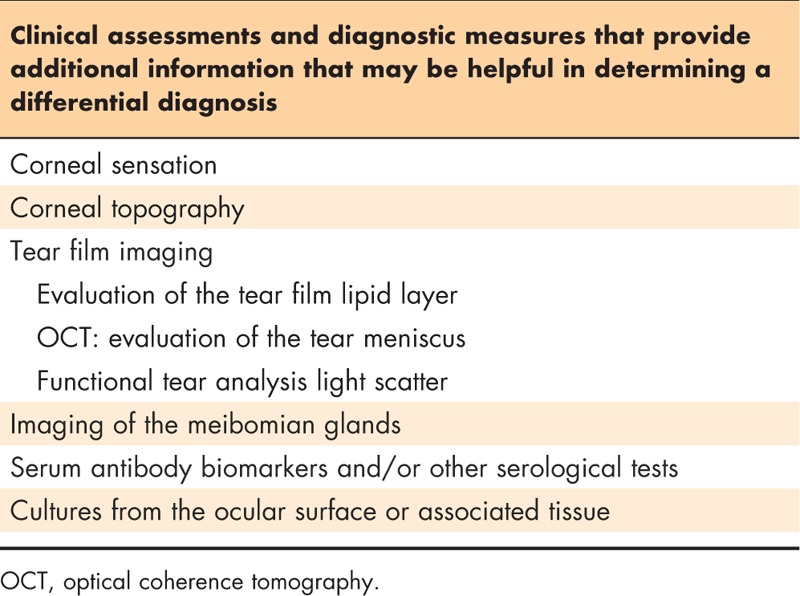
Additional assessments and diagnostic procedures

In conjunction with the patient's medical history and clinical findings, serologic testing provides insight or confirmation of a diagnosis of systemic conditions, such as Sjögren syndrome, through the presence of autoantibody biomarkers. Cultures and samples from the ocular surface or adnexa can be sent for laboratory analysis or testing onsite to investigate the presence of pathogenic organisms or elevated markers for other ocular conditions [e.g., immunoglobulin E (IgE) and lactoferrin]. The ‘Diagnostics and clinical assessments’ section presents additional detailed information pertaining to the description and interpretation of the diagnostic tests described previously.

### Step 5: differential diagnosis

Dysfunction of the tear film is a common condition that causes patients to seek ophthalmic care. ‘Dry eye’ is a term used to discuss a lack of tear production; however, the term is often used to refer to any tear disorder. Dry eye, a common cause of DTS, is currently estimated to affect 2–14.4% of the US population [22]. Extensive research has been conducted in the area of DED over the past several decades. The definition of DED has evolved based on understanding of the pathogenesis of the condition. The DEWS 2007 report [4] defined dry eye as ‘a multifactorial disease of the tears and ocular surface that results in symptoms of discomfort, visual disturbance, and tear film instability with potential damage to the ocular surface; it is accompanied by increased osmolarity of the tear film and inflammation of the ocular surface’.

The prevalence of blepharitis/MGD (evaporative and nonevaporative), another common cause of DTS that frequently alters the functionality of the tear film, has also been difficult to accurately determine. The results of recent surveys conducted among ophthalmologists and optometrists indicate that blepharitis has been observed in 37–47% of patients [23]. Additionally, a recent assessment of patients preparing to undergo cataract surgery indicated that 59% of patients were diagnosed with blepharitis [24]. MGD is a common cause of excess evaporation of the tear film, resulting in dry eye. Analysis of the frequency of distribution of dry eye subtypes indicates that MGD is one of the most common underlying conditions, with signs of MGD present in 86% of a mixed patient population with dry eye [25].

To achieve the primary goal of the DTS Panel of using a diagnosis-based approach to improve patient outcomes, all the evidence from the patient's medical history information, reported symptoms and effect on daily activities, clinical assessments, and diagnostics must be evaluated. A patient diagnosed with DTS can be classified with one or more of the four main disease-state subtypes described subsequently. Table 5 describes the primary characteristics and clinical findings that will aid in the identification of the DTS subtype(s) present in a particular patient. Additionally, it is important to recognize and treat any DTS co-conspirator conditions that may be masquerading as DTS or exacerbating a patient's signs and symptoms.

**Table 5:**
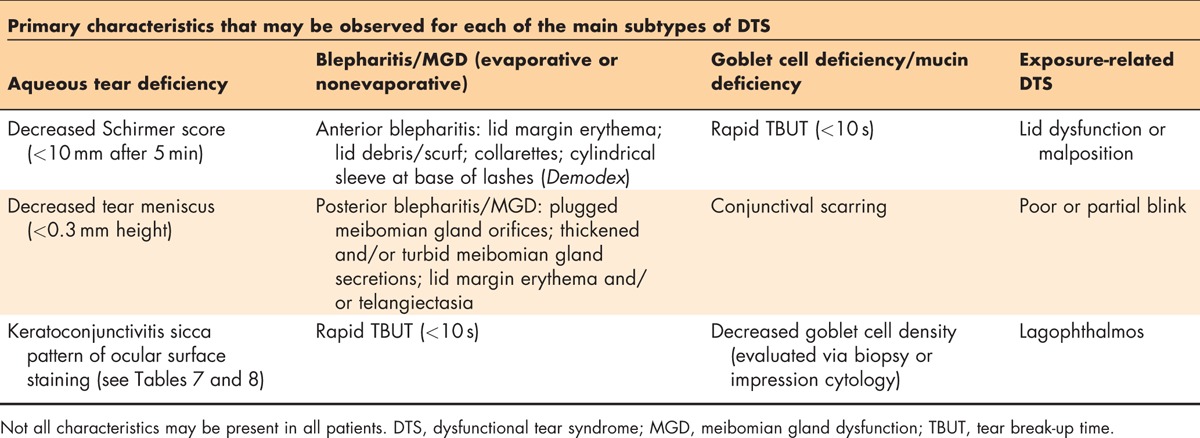
Differential diagnosis

#### Aqueous deficiency

Aqueous tear deficiency is primarily identified as a volumetric condition. Aqueous-deficient dry eye (ADDE) is one of the primary subtypes of DED, as recognized by the etiologic classifications system presented in the DEWS report [4]. Aqueous deficiency is characterized by a reduction in the secretions from the lacrimal gland (main and accessory glands) that form the basis of the bulk aqueous component of the tear film. The reduction in the aqueous secretions may be due to dysfunction or destruction of the lacrimal gland or a scarring/blockage of the lacrimal gland ductules, which prohibits the aqueous secretions from reaching the ocular surface, generally resulting in a reduced tear lake and elevated tear film osmolarity, or inflammation [26–28]. Because of the regulatory neuronal control of tear secretion, neurogenic inflammation – associated with injury, surgery, systemic conditions, and topical agents that decrease corneal sensation – may result in reduced activity of the lacrimal gland [4,22]. A further subclassification of ADDE is defined according to the presence or absence of a diagnosis of the patient with Sjögren syndrome [29–31].

Patients classified with aqueous deficiency generally have a reduced Schirmer score of less than 10 mm on the test strip. A score of less than 5 mm is not uncommon in patients with moderate-to-severe aqueous deficiency [19]. During the examination, a decreased tear meniscus is typically observed in patients with aqueous deficiency. Upon application of fluorescein and/or lissamine green/rose bengal dye to the ocular surface, a staining pattern characteristic of keratoconjunctivitis sicca is often present. The extent of staining will generally correlate with the severity of the aqueous deficiency [15,32].

#### Blepharitis/meibomian gland dysfunction (evaporative and nonevaporative)

As with DED, extensive basic and clinical research has been conducted in an effort to understand the pathogenesis of the various forms of blepharitis, particularly MGD. In a 2011 published report [5], the International Workshop on Meibomian Gland Dysfunction defined MGD as ‘a common disorder that may be asymptomatic or give rise to symptoms, either confined to the affected lids, or arising from MGD-related ocular surface disease, including evaporative dry eye (EDE); it can also exacerbate ADDE’.

Blepharitis is a condition that typically involves chronic inflammation of the eyelids, particularly the eyelid margin. The subtypes of blepharitis have been classified via a number of systems. Classification based on anatomic position, relative to the eyelid margin, includes anterior blepharitis affecting the skin of the eyelid, eyelashes, and eyelash follicles and posterior blepharitis affecting the meibomian glands and meibomian gland orifices. The clinical classification of anterior blepharitis includes the categories seborrheic, atopic, staphylococcal, herpetic, parasitic (i.e., *Demodex*), and fungal. As with other subtypes of DTS, combinations of the subtypes of blepharitis may occur simultaneously in patients [33,34]. The overgrowth of staphylococcal or other bacteria on the anterior portion of the lid margin may result in erythema, scaling, and crusting, with folliculitis, collarettes, or pustules present at the base of the lashes [33]. Several forms of anterior blepharitis are identified by greasy scaling/scurf around the lid margin for seborrheic, and sleeves at the base of lashes for *Demodex*. Other forms may be identified via culture [7].

Evaporative dry eye is the second primary subtype of DED, as defined by the etiologic classification system presented in the DEWS report [4]. Estimates indicate that EDE is the most common form of DED [25]. Excessive loss of the bulk aqueous component of the tear film (normally) secreted by the lacrimal gland, due to an insufficient or abnormal outer lipid component of the tear film, is the primary characteristic of evaporative tear disease [4]. Meibum, which is released from the meibomian glands present in the upper and lower lids and assisted by the blinking process, creates a thin oily layer over the bulk aqueous aspect of the tear film, thereby reducing evaporation. A lack of even distribution of the tear film due to insufficient production of soluble and membrane-bound mucins [35] or alterations in the nature or quality of meibum may result in a rapid TBUT, exposing the ocular surface to desiccating stress [36–38].

The outermost component of the tear film, the lipid layer, plays a critical role in the stability of the tear film. Alterations in the posterior lid margin, including the development of prominent blood vessels or telangiectasia, thickened or turbid secretions of the meibomian glands, and plugged gland orifices, are primary characteristics of MGD [7,39]. Changes in the tear film lipids or meibomian glands observed through interferometry and meibography can also provide valuable insight into the differential diagnosis of blepharitis/MGD. A focused examination of the anterior and posterior portions of the lid margin and manual expression of meibomian glands are important components for diagnosing blepharitis/MGD [7]. Although EDE associated with MGD is a common condition [25], the blepharitis/MGD subtype of the DTS Panel approach includes both evaporative and nonevaporative manifestations of the condition.

#### Goblet cell deficiency/mucin deficiency

Patients with goblet cell loss suffer from a subsequent reduction in mucin production. Attached mucin glycoproteins at the cell surface and soluble mucins interact with the aqueous component to affect the surface tension of the tear film and improve the spreading of tears across the ocular surface. Goblet cell loss and/or mucin deficiency affects the stability of the tear film. Although a rapid TBUT (<10 s) is frequently observed in patients with MGD, rapid TBUT is also observed in patients with goblet cell loss and/or mucin deficiency [40–42].

The primary characteristics of patients with goblet cell loss are a recognizable deficiency in goblet cell density (observed directly via impression cytology or conjunctival biopsy or inferred through observation of conjunctival inflammation and scarring [42]) and subsequent deficiency of mucin production. If available, in-vivo confocal microscopy can also be used to evaluate goblet cells and inflammation of the conjunctiva [43]. Goblet cell deficiency may result from or be associated with cicatricial conjunctivitis, such as Stevens–Johnson syndrome, toxic epidermal necrolysis, pemphigoid, thermal and chemical injuries, vitamin A deficiency, contact lens wear, and even epidemic keratoconjunctivitis (EKC). Additionally, patients with chronic chemical exposure or who habitually administer multiple ocular medications, such as glaucoma drops, may experience goblet cell loss [4,42,44–47].

#### Exposure-related dysfunctional tear syndrome

Excessive drying of the ocular surface due to anatomic defects, improper functioning, or malposition of the eyelids may result in exposure-related DTS. Failure of the eyelids to fully close or abnormal lid positioning exposes portions of the cornea to the external environment for an extended duration [48]. Exposure of the ocular surface beyond the interblink interval can initiate or exacerbate dysfunction of the tear film [49,50].

Observation of the positioning of the lids during the external examination, in conjunction with a careful review of the patient's medical history and chief complaints, can assist in a differential diagnosis of exposure keratopathy. Patients with Bell palsy, Parkinson disease, or other neurologic disorders may exhibit an incomplete or partial blink. Additionally, rigid contact lens wearers may have a reduced blink reflex in an effort to avoid disrupting the lens position. Characteristic staining patterns on the ocular surface are observed when dye is applied during a slit-lamp examination, thereby assisting in the diagnosis of exposure-related DTS due to conditions such as lagophthalmos. Lagophthalmos may be associated with complications resulting from blepharoplasty, scarring of the eyelid, thyroid eye disease, and other conditions [42,51].

#### Dysfunctional tear syndrome co-conspirators

The term ‘DTS co-conspirators’ is proposed to refer to conditions affecting the tear film and ocular surface that may masquerade or exacerbate DTS. DTS co-conspirators include superior limbic keratoconjunctivitis (SLK), medicamentosa, Thygeson superficial punctate keratitis, mucus fishing syndrome, contact lens-related toxicity, chemical toxicity, allergic/atopic conjunctivitis, conjunctivochalasis, floppy lid syndrome, and corneal hyperalgesia. Ocular allergy is a common DTS co-conspirator that often shares similar signs and symptoms with DED. Testing, such as in-office skin testing and tear film IgE analysis, may help identify this condition [52,53]. A thorough patient history review and examination are required to differentiate these DTS co-conspirators from one or more of the four main subtypes of DTS. Identification of the DTS co-conspirators is critical in the management of DTS because patients often present with persistent signs and symptoms despite general treatment for one or more of the subtypes of DTS.

Undiagnosed and untreated DTS co-conspirators can cause exacerbation of DTS and/or misdiagnosis because of the underlying condition. Other diseases that affect the ocular surface and tear film, such as allergic conjunctivitis, frequently coexist in patients with DTS and contribute to the signs and symptoms. All identified DTS co-conspirators should be addressed as part of a patient's treatment regimen. Treatment of these other ocular conditions is outside the scope of this monograph and should be managed according to the clinician's preferred treatment options. The ‘Diagnostics and clinical assessments’ section presents further discussion and detailed information pertaining to the description and interpretation of the diagnostic tests that are used to obtain a differential diagnosis.

### Step 6: treatment options

Once a differential diagnosis has been attained, including identification of all subtypes of DTS and/or DTS co-conspirators that may be present, a directed treatment regimen should be implemented to address the patient's condition. Table 6 summarizes the treatment options recommended by the DTS Panel for the four main subtypes of DTS. First-line therapy, second-line therapy, and interventional procedures are presented for each subtype of DTS, as appropriate. Inflammation and a reduced quantity or quality of the tear film is central to the various subtypes of DTS. As such, some of the recommended treatment options are included for multiple categories.

**Table 6:**
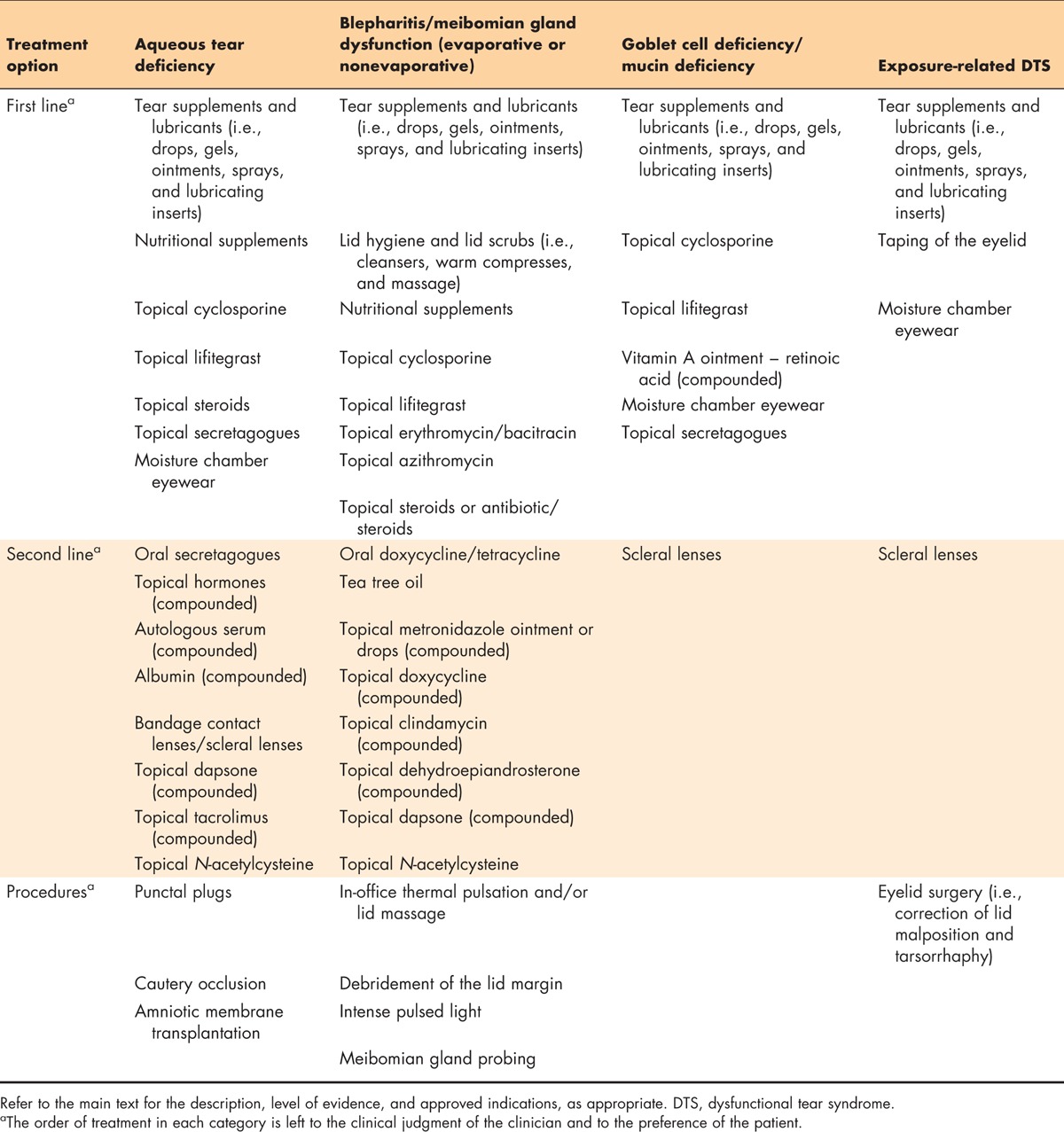
Recommended treatment options for the DTS Panel clinical approach

Artificial tears, gels, ointments, and inserts are used to replenish the tear film. Tears can be conserved via punctal plugs, cautery, and moisture chamber eyewear. Alterations in environmental conditions may benefit patients with DTS through increases in relative humidity. Adjustments in lid hygiene, warm compresses, and massage may also be necessary for patients with blepharitis/MGD. Anti-inflammatory and immunomodulatory agent options include cyclosporine, lifitegrast, steroids, and nutritional supplements, such as omega-3 fatty acids. In addition to the conventional treatments for DTS, innovative treatment options, including compounded formulations of agents, are also available.

The overall severity of a patient's DTS is an important factor that affects the sequence and frequency of the treatment regimen. The particular sequence for first-line therapy, second-line therapy, and interventional procedures is a decision best left to the individual clinician according to the patient's medical history, prior treatment, and severity of the current disease state. The ‘Treatment options for dysfunctional tear syndrome’ section presents a detailed discussion of each of the treatment options for the subtypes of DTS.

### Step 7: follow-up

Patient follow-up via regular clinic visits is an essential part of successful treatment of DTS. The fluctuations that occur in patient symptoms, signs, and diagnostic test results require the clinician to observe the patient over a period and to tailor treatment on the basis of response to therapy and changes from baseline values. Observed trends in inflammatory markers or tear film osmolarity provide a quantitative assessment of the progress of treatment from both the clinician and patient perspectives. The chronic nature of DTS and the frequent lack of correlation of clinical signs and patient symptoms emphasize the importance of monitoring a patient over time and making adjustments to the treatment plan, as necessary.

## DIAGNOSTICS AND CLINICAL ASSESSMENTS

A wide range of clinical assessments and diagnostic measures can be employed in the diagnosis of DTS. No single assessment or diagnostic procedure is sufficient for a differential diagnosis of DTS [6]. Identification of the specific subtypes of DTS is essential to determine a directed treatment approach. A thorough review of the patient's medical history, including past and current DTS symptoms and treatment, provides the context for interpretation of the current examination and diagnostic test results. This section presents the diagnostic tools that can be used to identify subtypes of DTS. Equipment access at each clinic and the preferences of each clinician differ and will influence the chosen diagnostic methods.

### Questionnaires

Patient-reported symptoms and the effects of DTS on daily activities can provide important information pertaining to the disease-state subtype and the severity of the condition. Although the clinically observed signs and patient-reported symptoms of DTS do not often correlate, addressing patient discomfort or functional limitations due to symptoms is a significant factor in determining the treatment approach. Validated questionnaire instruments designed with ranked scoring systems allow for quantification of patient responses and an overall estimate of the severity of their discomfort and the effect on visual function [4]. Patient responses to individual questions contribute to the overall score in the assessment and may also help direct the diagnosis of the particular DTS subtype.

A variety of questionnaires to evaluate the symptoms of dry eye have been developed [4]. Questionnaires such as the Ocular Surface Disease Index (OSDI) [54] and the Standard Patient Evaluation of Eye Dryness [55] are commonly used in standard clinical practice to assess patient symptoms and in clinical trial research to screen potential individuals or to evaluate the effect of treatment on individual-reported symptoms and visual function [54].

### Slit-lamp examination

**FIGURE 3:**
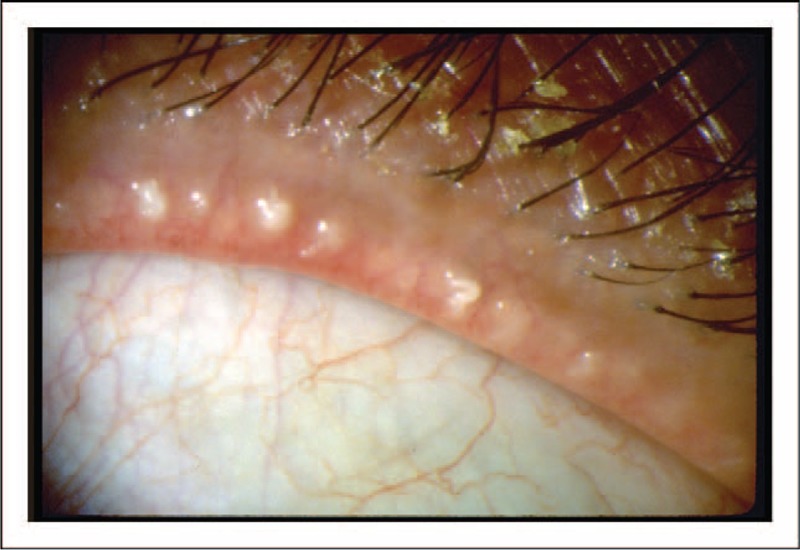
Meibomian gland orifices and expression of the meibomian glands. Image from a patient with posterior blepharitis/meibomian gland dysfunction (MGD). Obstruction of the orifices of the meibomian glands and alterations in the meibum, leading to turbid, thickened secretions, can occur in patients with blepharitis/MGD. © 1994 American Academy of Ophthalmology.

Detection of abnormalities affecting the eyelid, anterior and posterior lid margins, conjunctiva, and cornea observed during the slit-lamp examination offers insight toward a differential diagnosis of DTS subtypes and identification of DTS co-conspirators that may exacerbate a patient's DTS condition [6]. During the initial portion of the slit-lamp examination, the following should be observed in detail:

(1) Tear film meniscus (tear lake) and any debris or foam that may be present in the tear film
(2) Lashes for any abnormalities (i.e., loss, misdirection, sleeves, scurf, and collarettes)
(3) Anterior and posterior lid margins, noting the characterization of the vasculature and any abnormalities in the meibomian gland orifices
(4) Nature of the meibomian gland secretions – grade 0–3 scale: 0 = clear/normal; 1 = cloudy; 2 = cloudy particulate fluid; and 3 = inspissated (like toothpaste) [5] (Fig. 3)
(5) Appearance of the puncta
(6) Any anomalies of the conjunctiva and cornea (i.e., hyperemia, mucus strands, scarring, and epithelial erosions) [5]

Each abnormality may be ranked as mild, moderate, severe, or according to some other system to allow for a baseline comparison at follow-up visits.

**FIGURE 4:**
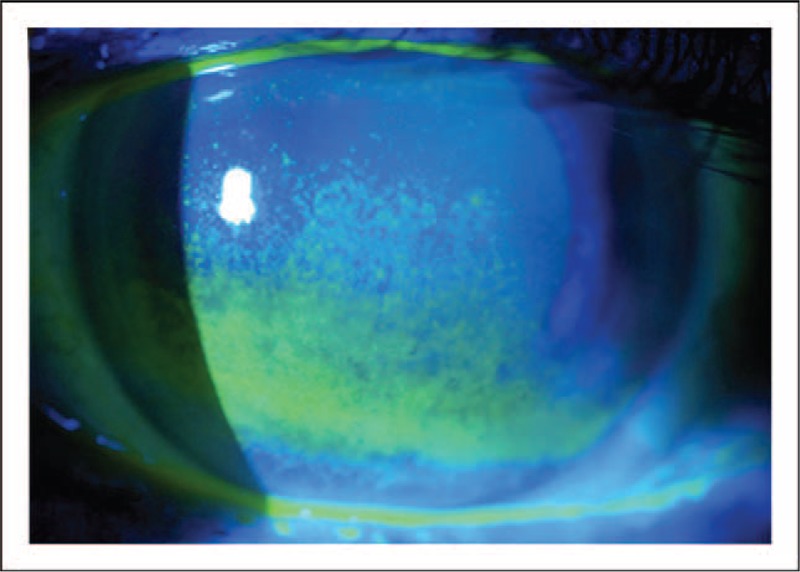
Corneal fluorescein staining. Image from a patient with aqueous deficiency. The disruption in the integrity of the corneal epithelium is highlighted by the application of fluorescein staining to the ocular surface. Moderate-to-severe staining with fluorescein is depicted in the photograph. Image courtesy of Karl Stonecipher, MD.

Once the initial examination is complete, dye is introduced on the ocular surface to further evaluate the tear film and corneal/conjunctival tissue. Instillation of liquid sodium fluorescein or application of fluorescein dye via moistened strips to the ocular surface allows for visualization of the tear film and integrity of the corneal and conjunctival epithelium (Fig. 4). Fluorescein dye will penetrate tissue where the epithelial intracellular junctions have been disrupted (i.e., cells that are dead or absent). The stability of the tear film can be observed via the slit lamp with a cobalt blue filter following application of fluorescein. To evaluate the stability of the tear film, the TBUT test is usually conducted before any other drops or anesthetic are applied. The patient is instructed to blink a few times, and the time interval between blinking and disruption of the tear film is measured several times for each eye (averaging the time for each eye). A rapid TBUT (<10 s) is considered abnormal and often indicates evaporative tear disease/MGD and/or aqueous tear deficiency [6,19].

During evaluation of TBUT, the pattern of the tear film disruption may offer insight into the nature of the damage to the ocular surface tissue. In addition to the presence or absence of staining of the ocular surface, the severity and pattern of the staining may assist in the differential diagnosis of DTS or DTS co-conspirators. Evaluating patients across multiple visits with consistent application of the tests will allow for trends to be observed, which help overcome the fluctuation in clinical signs often observed in patients with DTS.

**FIGURE 5:**
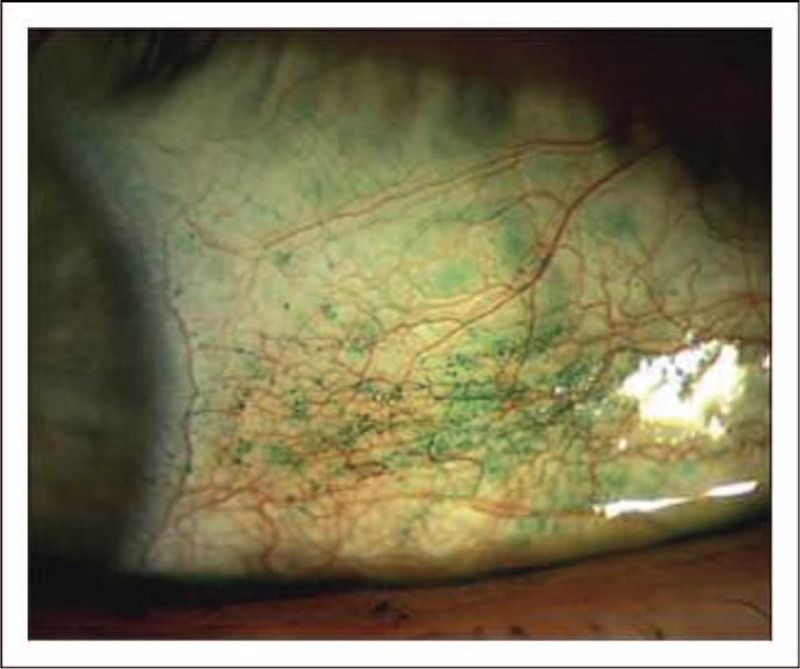
Staining of the conjunctiva with lissamine green. Image from a patient with aqueous deficiency. Vital dyes, such as lissamine green, may be used to visualize debris in the tear film and regions of the conjunctiva that are deficient in mucin. Moderate-to-severe lissamine green staining of the temporal aspect of the conjunctiva is shown. Image courtesy of Elizabeth Yeu, MD.

**FIGURE 6:**
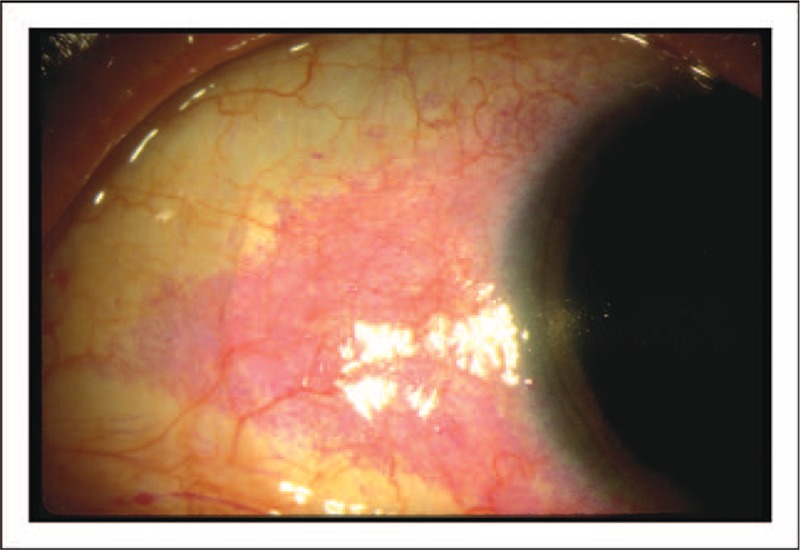
Staining of the conjunctiva with rose bengal. Image from a patient with aqueous deficiency. Rose bengal may be used to highlight areas of the conjunctiva that are abnormal or unhealthy in patients with dysfunctional tear syndrome (DTS). Moderate staining of the conjunctiva is shown with a classic pattern for keratoconjunctivitis sicca. © 1994 American Academy of Ophthalmology.

Table 7 presents corneal/conjunctival fluorescein staining patterns characteristically associated with a range of ocular conditions. Vital dyes, including lissamine green (Fig. 5) and rose bengal (rose bengal may be uncomfortable when applied to the ocular surface) (Fig. 6), are recommended to be applied to the ocular surface once the fluorescein evaluation is complete. Vital stains assist in the visualization of debris in the tear film and areas of the ocular surface that do not have a mucus coating (i.e., cells that are ‘unhealthy or abnormal’) [20,21,56]. Table 8 presents corneal/conjunctival lissamine green/rose bengal staining patterns characteristically associated with a range of ocular conditions. It is important to note that mild fluorescein staining may be present even in normal eyes [6]. Also, diagnostic procedures, such as the Schirmer test, may result in mild staining with fluorescein, rose bengal, or lissamine green in the region where the test strip was placed.

**Table 7:**
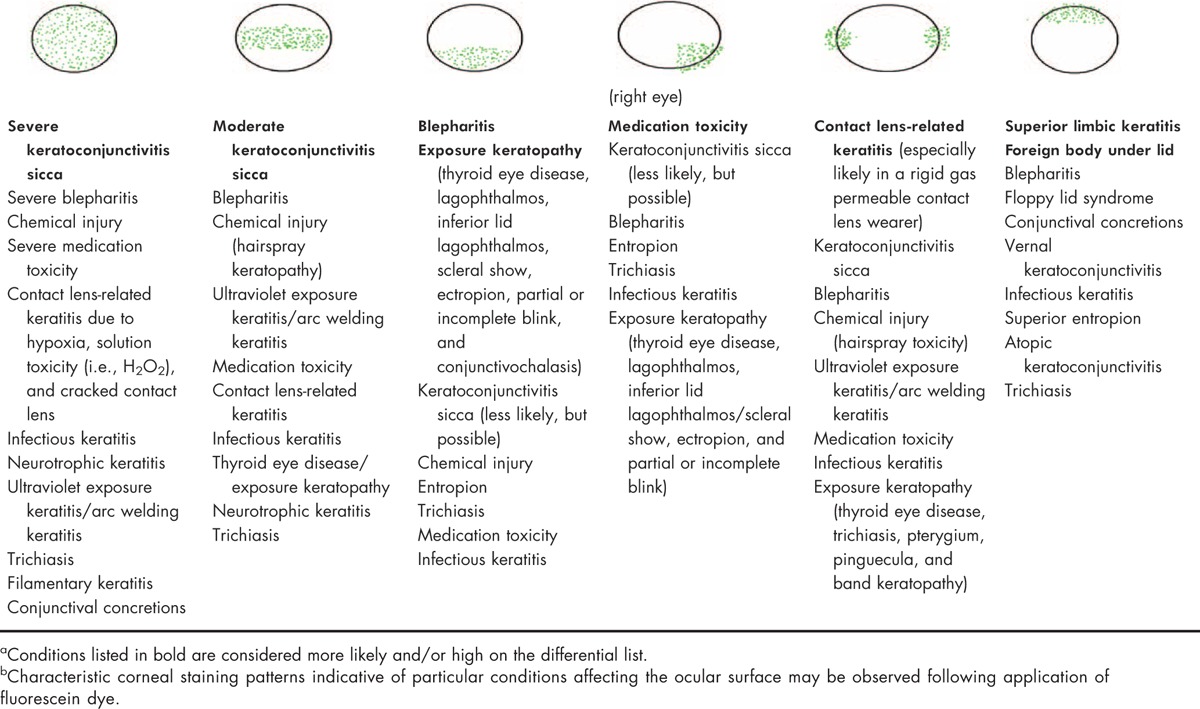
DTS Panel approach ocular surface fluorescein staining patterns^a^^,^^b^

**Table 8:**
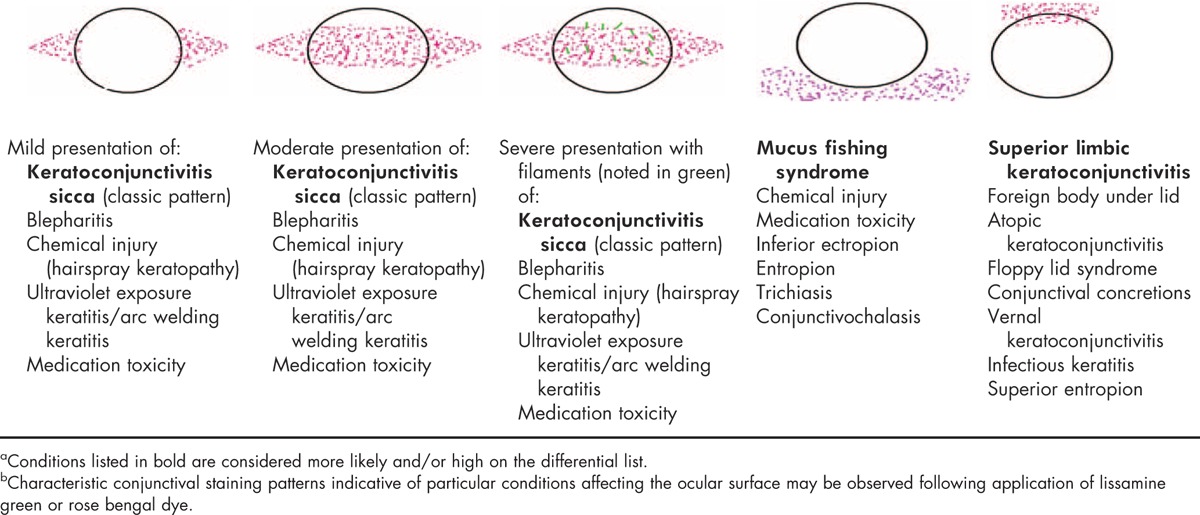
DTS Panel approach ocular surface lissamine green/rose bengal staining patterns^a^^,^^b^

Scoring systems for ocular surface staining have been developed in an effort to standardize patient assessments for clinical practice and research. Adoption of a scoring system is not necessary for use of the DTS Panel approach to arrive at an accurate diagnosis; however, clinicians may find the use of a scoring system (e.g., National Eye Institute scale or Oxford scheme) helpful in monitoring a patient's response to treatment [30,57,58].

### Schirmer test

**FIGURE 7:**
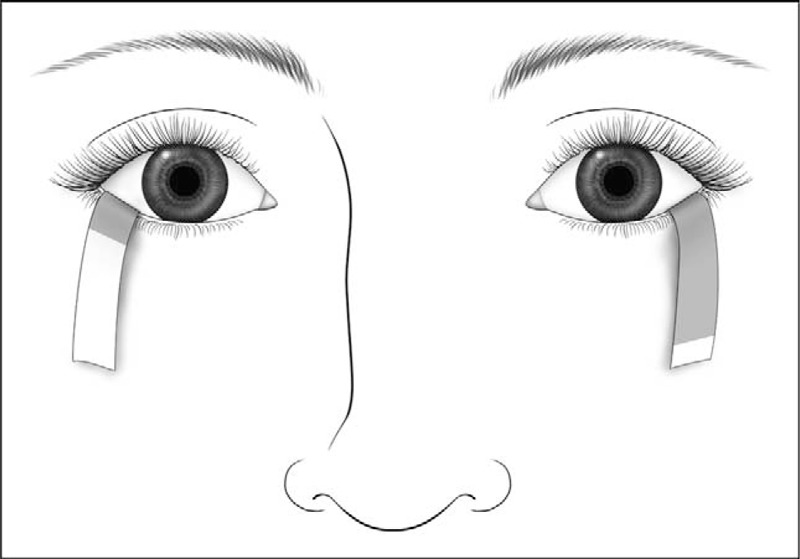
Schirmer strip test. Illustration of a patient undergoing Schirmer strip testing. Abnormalities in the production of the aqueous component of the tear film may be diagnosed using Schirmer strip testing. The Schirmer strip is placed in the inferior cul-de-sac near the lateral canthus.

The Schirmer test is a diagnostic method for evaluating aqueous tear production [19,59]. Clinicians use several variations of this procedure. Topical anesthesia may or may not be used during the procedure, and, in keeping with clinician preference, the procedure may include nasal stimulation to induce reflex tearing. The use of topical anesthesia prior to the assessment allows for the measurement of basal tear secretion, as compared with basal plus reflex tearing from the nonanesthetized version of the test. To perform the test, a narrow strip of filter paper (Schirmer strip) is placed in the inferior cul-de-sac near the lateral canthus (Fig. 7). Excess fluid in the fornix should be dried prior to insertion of the test strip. The length of the test strip that is wetted after 5 min is measured in millimeters. A Schirmer test score of less than 10 mm after 5 min is generally considered abnormal [6,15].

The phenol red thread test is an alternative method to evaluate tear volume. The thread is placed in the inferior cul-de-sac toward the lateral canthus [3]. The length of the thread wetted (indicated by a color change) in 15 s is measured and recorded. A length of less than 10 mm of the thread after 15 s is considered abnormal.

Although the Schirmer test is frequently used in the diagnosis of various subtypes of dry eye, the results of the test are often variable [60]. Single abnormal test results may not be considered sufficient for a diagnosis. As with other diagnostic procedures used to assess patients with potential DTS, the observation of trends indicating consistently abnormal findings are beneficial in determining a differential diagnosis [6,15].

Members of the DTS Panel recommend the use of the Schirmer test as part of the battery of diagnostic assessments. Panel members use the test on both anesthetized and nonanesthetized patients. Both methods of employing the tests are correct and provide insight into the patient's aqueous tear production capacity. Additionally, a Schirmer test assessment should be considered for patients with DTS prior to punctal plug insertion to decrease the risk of secondary epiphora.

### Tear film osmolarity

Osmolarity is a measurement of the concentration of dissolved solutes in a solution. In terms of the tear film, osmolarity is generally expressed in units of milliosmoles per liter. Hyperosmolarity of the tear film is a recognized and validated marker of dry eye [13]. Hyperosmolarity of the tear film occurs through decreased flow of the bulk aqueous component of the tear film from the lacrimal gland and/or through increased evaporation and instability of the tear film. Increased osmolarity of the tear film stimulates the release of inflammatory cytokines, enhances the rate of cell apoptosis, and results in a decrease in the number of goblet cells [12,13].

Patients with a normal tear film typically have a stable tear film osmolarity. A higher degree of fluctuation in tear film osmolarity is observed in patients with DTS. Fluctuations occur both between measurements taken from the same eye and measurements concurrently taken between the eyes of a patient. The inherent fluctuations in tear film osmolarity of patients with DTS can be a point of confusion for clinicians unless the instability of the tear film is recognized as a hallmark of DED. A point-of-care test for the measurement of the osmolarity of the tear film of patients suspected of having DTS is available [14]. In general, an elevated tear film osmolarity correlates with DTS. The normal tear film osmolarity in patients without DTS ranges from 270 to 308 mOsm/l (mean of 302 mOsm/l). A threshold of 308 mOsm/l has been found to be indicative of early/mild dry eye, whereas a tear film osmolarity of 316 mOsm/l or higher is correlated with moderate-to-severe dry eye. Mean tear film osmolarity values of patients with mild-to-moderate dry eye are 315 mOsm/l, whereas those of patients with severe dry eye have been found to be 336 mOsm/l. Based on the stability of the tear film in the eyes of normal patients, a difference in the tear film osmolarity of more than 8 mOsm/l between the eyes is suggestive of ocular surface instability and indicative of DED [12–14]. In addition to the specific tear osmolarity values associated with the severity of DED listed previously, clinicians may also find it useful to generally categorize the assessed severity level using ranges of up to 300 mOsm/l for normal, 300–320 mOsm/l for mild, 320–340 mOsm/l for moderate, and above 340 mOsm/l for severe DED [61].

Evaluation of tear film osmolarity should be conducted prior to disturbance of the eye to obtain accurate results because any disturbance to the ocular surface may stimulate reflex tearing, which can falsely lower the osmolarity reading [62]. Although an elevated measurement of tear film osmolarity is a strong indicator of DTS, the presence of an elevated osmolarity reading may not correlate with a patient's symptoms or other clinical signs [62] because of the nature of the disease and fluctuations observed in these patients. Trends observed through multiple osmolarity assessments are beneficial to increase the sensitivity and specificity of the diagnosis of individual patients. If used, tear film osmolarity should be assessed at the initial evaluation of a patient with DTS and at follow-up visits to establish a baseline and quantify the patient's response to treatment as the tear film stabilizes. The tear film osmolarity assessment has been demonstrated to have an 88% specificity and 78% sensitivity for mild/moderate dry eye and a 95% sensitivity for severe dry eye [6,13,14]. Despite these results, some studies question the accuracy of the tear film osmolarity assessment and have shown variability in the osmolarity measurements, with a lack of strong correlations with other signs and symptoms of DED [63–66]. Bunya *et al*. [63] suggest that tear film osmolarity testing has feasibility in the clinical setting and induces less discomfort than the Schirmer test. On the basis of their experience, members of the DTS Panel have found the assessment of tear osmolarity helpful for diagnosing DTS when used in combination with other clinical assessments and procedures.

### Inflammatory markers in the tear film

Inflammation is a common factor across the subtypes of DTS. The inflammatory cascade responsible for producing inflammation on the ocular surface in DTS offers the opportunity to correlate measurements of inflammatory biomarkers with the severity of the disease. The levels of inflammatory mediators, including cytokines, chemokines, and enzymes, involved in tissue remodeling may be assessed in the tear film. Recent research has evaluated a member of the MMP family, MMP-9, an enzyme produced by corneal epithelial cells, as a biomarker for dry eye [16,17]. The MMP family of enzymes plays an important role in wound healing and inflammation through the ability to degrade collagen. Elevated levels of MMP-9 have been observed in the tears of patients with dry eye [67].

The normal range of MMP-9 in the tears of patients ranges from 3 to 40 ng/ml. In contrast, the MMP-9 levels in the tears of patients with moderate-to-severe DTS can exceed 40 ng/ml. A point-of-care test for the measurement of the concentration of MMP-9 in the tear film of patients suspected of DTS is available [18,67]. Because of a requirement for MMP-9 levels to be greater than 40 ng/ml to provide a positive assessment using the point-of-care diagnostic, the use of MMP-9 as a diagnostic measure may be valid only for patients with more severe forms of DTS [68].

### Corneal sensation

The cornea, one of the most highly innervated tissues in the body, is susceptible to alterations in sensitivity because of damage or injury. Irritation or injury to the ocular surface due to hyperosmolarity or inflammation triggers the reflex arc to stimulate the lacrimal gland secretions. Changes in the sensitivity of the cornea have been observed in patients with chronic conditions such as DTS. Sensitivity may be increased during the early stages of the disease, whereas a reduction in corneal sensitivity is associated with disease progression [19,69,70].

Instruments to precisely quantify corneal sensitivity, such as the Cochet–Bonnet esthesiometer, are available and used in basic research or clinical trial settings [71]. In general clinical practice, access to esthesiometers may be limited; consequently, qualitative methods can be employed to assess changes in a patient's corneal sensitivity. Basic scoring systems may be developed through the use of simple tests for sensation, in which a cotton swab, unwaxed dental floss, or the tip of a tissue is gently applied to the ocular surface. In the authors’ clinical experience, grading scales, including numeric scales (0–4, ranging from no sensation to normal sensation exhibited via a reflex to pull away) or descriptive scales (normal, decreased, or none), may be used to characterize corneal sensation.

### Lacrimal gland secretion biomarkers

The secretions of the lacrimal gland that form the bulk of the aqueous component of the tear film contain a range of proteins, including enzymes and immunoglobulins, electrolytes, and other factors involved in maintaining the health of the ocular surface. Assessments of the protein components of lacrimal secretions have found altered levels in the tear film of patients with diseases affecting the ocular surface, which allows these proteins to be used as biomarkers [72,73].

Lactoferrin, a molecule belonging to the transferrin class of proteins, is one of the most abundant protein components of the healthy tear film. Through the sequestration of iron, lactoferrin acts as an antimicrobial agent and plays a role in the immunologic and anti-inflammatory properties of the tear film. The concentration and absolute protein levels of lactoferrin in the tear film have been observed to be reduced in patients with aqueous-deficient dry eye [74]. A point-of-care test for the measurement of the concentration of lactoferrin in the tear film of patients suspected of having DTS is available. Lactoferrin levels below 0.9 mg/ml suggest the patient has ADDE, with the severity of the disease correlating with lower levels of the biomarker [72,73].

The immunoglobulin family of proteins plays an essential role in the functioning of the immune system. IgE proteins, antibodies specific for particular allergens, are found in the tear film of patients with ocular allergic conditions. Exposure to allergen particles results in binding of IgE and interaction with mast cells in the conjunctiva, initiating the inflammatory response of the allergic cascade [75,76]. A commercially available point-of-care test designed to evaluate the level of IgE in tear samples with IgE levels at least 80 ng/ml suggests a diagnosis of allergic conjunctivitis, with the level of IgE present in the tear film correlating with the severity of the allergic condition [53].

### Optical coherence tomography

**FIGURE 8:**
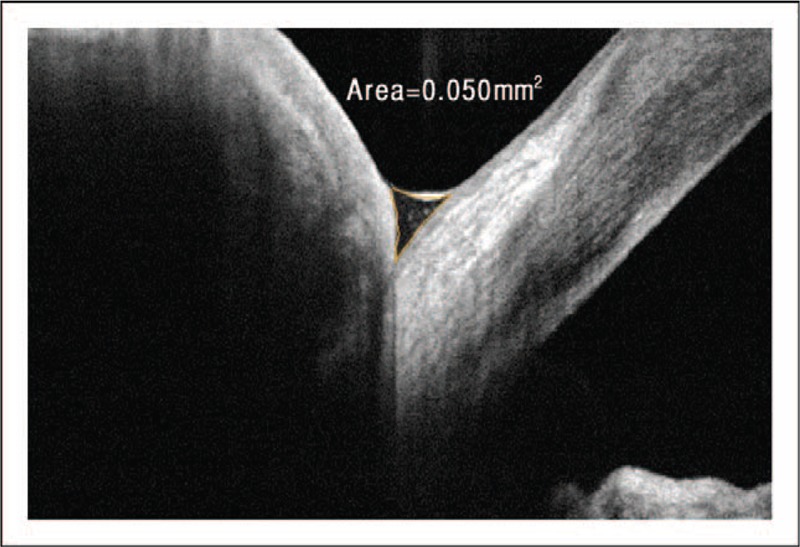
Evaluation of the tear film meniscus with optical coherence tomography (OCT). Image of the cross-sectional area of the tear film meniscus with OCT. Evaluation of the tear film with a high-resolution OCT allows for the characteristics of the tear film to be quantified. The area of the inferior meniscus was calculated to be 0.05 mm^2^ in this patient, which was within the normal range [77]. Image courtesy of Elizabeth Yeu, MD.

OCT is a noninvasive technique that uses light waves to construct a three-dimensional, high-resolution, cross-sectional image of biologic tissues or fluids. OCT devices capable of evaluating the anterior segment offer the opportunity to quantify aspects of the tear film. The precise height, volume, and cross-sectional area of the tear meniscus can be measured. Figure 8 presents a representative OCT image showing the cross-sectional area of a normal tear meniscus [77]. In addition to a comparison of the tear meniscus characteristics to the mean values of patients with DTS versus those without DTS, OCT offers a means to monitor a patient's response to treatment options. Improvements in the tear meniscus following the installation of punctal plugs or other therapy for DTS allow the clinician to provide quantitative feedback to patients [78,79]. Recent advances include the automation of analysis of the tear meniscus height over multiple image scans, which potentially increase the accuracy of the measurement compared with a single scan [80].

### Interferometry and meibography

Interferometry is a noninvasive technique that can be used to measure the thickness of the tear film through the use of the principle of optical interference or the interaction of light waves. Colored images of the superficial layer of the tear film, the lipid layer, can be generated and evaluated to assess the thickness across the ocular surface [15,81].

**FIGURE 9:**
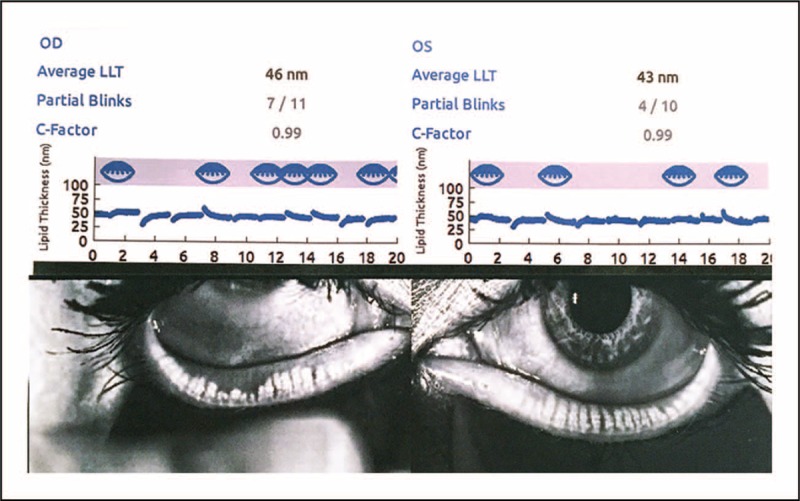
Visualization of the meibomian glands, tear film lipid layer, and partial blinking. Image of a patient with meibomian gland dysfunction (MGD) and exposure-related dysfunctional tear syndrome (DTS). Diagnostic systems designed for visualization of the meibomian gland (meibography) are also capable of providing an analysis of the thickness of the tear film lipid layer and an assessment of partial blinking. Note a decrease in lipid layer thickness of 46 nm in the right eye and 43 nm in the left eye; increased partial blinking OU; and meibomian gland truncation OD greater than OS. Image courtesy of Mark Milner, MD.

Diagnostic devices are commercially available, allowing for the visualization and assessment of a patient's tear film lipids [82,83]. A reduction in the thickness of the lipid component of the tear film (<60 nm) has been correlated with MGD and symptoms of dry eye [78]. Diagnostic systems also allow for the recording of a patient's blink cycle, thereby determining whether a full closure of the interpalpebral fissure is occurring [78]. Upgrading the diagnostic systems allows for visualization of the meibomian gland via meibography, further assisting in the differential diagnosis of MGD (Fig. 9). Meibography techniques may be used to visualize alterations in the meibomian glands, such as dropout, dilation, and truncation [83,84].

### Serologic biomarkers

Sjögren syndrome is a systemic autoimmune disease that often includes ADDE as an ocular manifestation. The salivary glands are affected in many patients, and symptoms of dry eye and dry mouth are considered hallmark indications of Sjögren syndrome. Serologic testing, confirming the presence of autoantibodies, including SS-A or SS-B, has been typically required to diagnose primary or secondary Sjögren syndrome. Novel antibodies that appear to be detectable early in the development of Sjögren syndrome have recently been identified [85]. A diagnostic panel that tests for autoantibodies to salivary protein-1, parotid secretory protein, and carbonic anhydrase VI, in addition to the traditional autoantibody markers for Sjögren syndrome, is available. The diagnostic panel uses a small blood sample from the patient, which is obtained by finger prick or venipuncture. The availability of this diagnostic panel, which contains early biomarkers for Sjögren syndrome, enables earlier detection and directed treatment of this progressive condition [85–87].

## TREATMENT OPTIONS FOR DYSFUNCTIONAL TEAR SYNDROME

A directed treatment plan based on a differential diagnosis is considered key to achieving the primary goal of the DTS Panel approach to improving patient outcomes. This section presents treatment options for the DTS clinical subtypes. Overlap of specific treatment options occurs between DTS subtypes, and, in such cases, the information presented reflects the published evidence or expert opinion supporting the use of a therapy for that particular condition.

Each section includes information pertaining to treatment options approved by the US Food and Drug Administration for a specific indication, as appropriate. This section also includes additional therapeutic options on the basis of a review of the published evidence and the expert opinion of the authors, in which evidence is limited. Information and recommendations considered ‘off-label’ are also included. As such, the decision and manner to use any therapy is best determined by each individual clinician's best judgment.

Multiple treatment options are presented for each subtype of DTS. Although the emphasis of this approach is to direct treatment on the basis of the specific disease-state subtype(s) for each patient, the relative severity of a patient's condition is also an important consideration in the sequence and/or combination of therapies applied in any treatment regimen.

Table 6 categorizes the treatment options in groups recommended for first-line therapy, second-line therapy, and interventional procedures. It is important to keep in mind, however, that each patient is unique, and the decision regarding which treatment option(s) to use is best left to the individual clinician. Treatment options for each DTS subtype are presented on the basis of treatment category rather than the order in Table 6.

Patient education regarding early diagnosis, risk factors, treatment options, and prognosis of DTS is considered a part of the first-line treatment plan for all subtypes of DTS. All patients may benefit from efforts to mitigate the severity and effect of DTS symptoms on quality of life through potential adjustments to their behavior, daily activities, and exposure to environmental stressors.

### Aqueous deficiency treatment options

#### Tear supplements and lubricants

Tear supplements and topical lubricants are available in a range of options for the treatment of ocular symptoms associated with DTS. Artificial tears serve to lubricate the ocular surface and remove debris, supplement-deficient components of the tear film, dilute a hyperosmolar tear film, and reduce elevated levels of proinflammatory mediators [4,5,88–91]. The current array of tear supplements and lubricants are distinguished by their formulation differences, which include variations inviscosity, electrolytes, pH, osmolarity, and the presence/type of a preservative [4].

Artificial tears are considered as first-line therapy for tear deficiency and other types of DTS because of availability, their noninvasive nature, and a generally minimal side-effect profile [6]. Clinical studies investigating the efficacy of various formulations of tear supplements have been conducted, including comparative studies to evaluate the differential effects on patient symptoms and clinical signs of DTS [92]. The use of preserved tear supplements is appropriate for many patients with mild tear deficiency to relieve the symptoms of DTS, as needed. However, because of the potential for disruption of the tear film and toxicity to the corneal epithelium, the authors recommend that patients with moderate-to-severe tear deficiency requiring the use of artificial tears more than four times per day use preservative-free formulations. The potential for ocular toxicity has been well characterized for benzalkonium chloride. Newer preservative agents may still potentially damage the corneal epithelium [92–95]. Preservative-free artificial tear supplements may be used liberally, as needed.

#### Lubricating inserts

Hydroxypropyl cellulose ophthalmic inserts are sterile, water soluble, and slow-release lubricants that are placed in the inferior cul-de-sac. Hydroxypropyl cellulose ophthalmic inserts are indicated for patients with moderate-to-severe dry eye. Because the formulation is preservative free, it may help patients who are not able or willing to use artificial tears on a frequent basis [96,97]. Published clinical evidence of efficacy (level II) has reported a significant improvement in tear deficiency symptoms and OSDI scores with once-daily use of hydroxypropyl cellulose ophthalmic inserts [98,99]. Transient blurred vision was the most common adverse event reported that was associated with use of the hydroxypropyl cellulose ophthalmic inserts [97,98].

#### Nutritional supplements

Essential fatty acids, including omega-3 and omega-6 fatty acids, must be obtained through dietary consumption. Omega-3 and omega-6 fatty acids are required for the normal function of human cellular metabolism. Dietary intake of essential fatty acids may play a role in the risk for developing subtypes of DTS. A diet that has a higher ratio of omega-6 fatty acids (a precursor for proinflammatory mediators) is associated with an increased risk of dry eye. Conversely, a diet that has a high ratio of omega-3 fatty acids is associated with a reduced chance of developing dry eye [100].

Evidence from clinical investigations involving dietary supplements, primarily omega-3 fatty acids, generally supports that intake is associated with an improvement in the symptoms of tear deficiency. Recent meta-analyses of randomized, placebo-controlled studies (level I) indicated that significant improvements in TBUT and Schirmer test scores were associated with daily dietary intake of omega-3 fatty acids (eicosapentaenoic acid and docosahexaenoic acid) [101]. Significant improvements in individual-reported symptoms (OSDI assessment) and objective evaluations of corneal smoothness were observed in a clinical evaluation of long-term consumption (6 months) of nutritional supplements containing gamma-linolenic acid and omega-3 fatty acids by patients with moderate-to-severe dry eye compared with ingestion of a placebo (level I). The study reported that no ocular adverse events were attributed to the nutritional supplement or to the placebo; similarly, no significant systemic adverse events were noted in either treatment group [102]. Omega-3 fatty acid supplements are available from a range of sources and formulations [101]. Clinical investigation regarding the recommended dosage and formulation of these supplements is ongoing.

#### Topical cyclosporine

Inflammation associated with the ocular surface and secretory components of the lacrimal system is believed to be a core feature of the pathogenesis of dry eye. Changes in the functioning of the lacrimal gland and/or lacrimal ducts may affect both the quantity and quality of the secretions of the lacrimal gland that form the bulk aqueous component of the tear film [103,104]. Inflammation associated with the ocular surface may be induced through chronic irritation due to environmental stress, ocular comorbidities, physiologic changes associated with aging, or ocular manifestations of systemic conditions. Therapy with anti-inflammatory agents is recommended because of the strong correlation of inflammation with tear deficiency [4,6,105,106].

Cyclosporine A, a peptide derived from a fungal origin, is an anti-inflammatory agent with immunosuppressive properties. Topical application of cyclosporine (0.05%) to the ocular surface is indicated to increase tear production in patients who have decreased tear production due to inflammation associated with dry eye [107]. The precise mechanism of action of topically applied cyclosporine is unknown; however, the agent is believed to act as a T-cell immunomodulator in patients with tear deficiency [107].

Phase 3 clinical studies supporting the approval of topical cyclosporine for the treatment of dry eye indicated a significant increase in Schirmer test scores in individuals treated with cyclosporine twice daily compared with those treated with vehicle (level I). A significant decrease in fluorescein corneal staining and improvement in subjective measures (e.g., blurred vision, need for artificial tear use, and global response to treatment) were also observed in the individuals treated with topical cyclosporine compared with those treated with vehicle [4,107,108]. An early response time ranging from 4 to 6 weeks to 3 months may be observed in patients, and benefits may continue to improve for 2 years or more. Clinical improvements in the signs and symptoms of DED were observed in patients with severe manifestations of the disease following high-frequency administration (three to four times daily) of topical cyclosporine (level II) [109]. Ocular burning and stinging were the most commonly reported adverse events in the individuals treated with topical cyclosporine [108–110]. The authors recommend a dosage regimen of twice-daily application of topical cyclosporine to both eyes in the treatment of tear deficiency for a minimum of 6 months [111]. Patients should be assessed at follow-up visits, and therapy is commonly maintained indefinitely for this chronic condition, as appropriate. According to the judgment of the clinician, if the patient's signs and symptoms have fully resolved, therapy with topical cyclosporine may be discontinued or reduced to once-daily administration following 1 year of twice-daily treatment. Clinical evidence indicates that once-daily administration following 1 year of twice-daily administration may still suppress the signs and symptoms of DED (level II) [112]. Members of the DTS Panel have used topical cyclosporine therapy for 2 years or more for patients with aqueous deficiency. Limited safety information is available from the published studies evaluating long-term and/or a reduced administration regimen of topical cyclosporine.

#### Topical lifitegrast

Activation and recruitment of immune cells, specifically lymphocytes, occur in the development and perpetuation of DTS through the binding of receptors on the surface of immune cells with molecules expressed on the epithelium of the ocular surface and vascular endothelium. Lymphocyte function-associated antigen-1 (LFA-1) is a member of the integrin receptor family that is expressed on lymphocytes and binds to adhesion molecules, such as intracellular adhesion molecule-1 (ICAM-1). Inhibition of the interaction between LFA-1 and ICAM-1 by the integrin receptor antagonist lifitegrast (SAR 1118) results in blocking the recruitment, activation, and release of proinflammatory mediators by lymphocytes [113–115].

Topical application of a lifitegrast ophthalmic solution (5.0%) is indicated for the treatment of the signs and symptoms of DED [116]. Clinical evaluation of topical lifitegrast in the treatment of individuals with dry eye (twice-daily administration) resulted in significant improvement in corneal and conjunctival staining as well as improvements in ocular symptoms, including eye dryness, compared with placebo [115,117,118]. Adverse events were generally mild to moderate and transient in nature, with the majority of ocular adverse events related to instillation in both treatment groups (e.g., reduced visual acuity, irritation, and discomfort), whereas dysgeusia was the most common nonocular adverse event reported by individuals treated with lifitegrast [115,118,119].

The approved dosage regimen of topical lifitegrast is twice-daily application to both eyes (OU) [116]. Patient response to treatment should be assessed at follow-up visits, and therapy may be maintained indefinitely, as appropriate.

#### Topical secretagogues

Secretagogues are agents that induce secretion as a mechanism of action. Application of secretagogues to the ocular surface are designed to stimulate aqueous and/or mucin secretion. Topical therapeutic agents in the secretagogue class have been investigated, but are not currently available in the United States [120,121]. Topical secretagogue formulations (e.g., 3% diquafosol sodium ophthalmic solution and 2% rebamipide ophthalmic suspension) are available internationally and indicated for the treatment of dry eye [122,123].

#### Topical steroids

Corticosteroids are anti-inflammatory compounds with use in treating a range of ocular inflammatory conditions. In the United States, topical ophthalmic formulations of corticosteroids are generally indicated for the treatment of steroid-responsive inflammatory conditions affecting the anterior segment. Clinical evidence from multiple studies using different formulations indicates that corticosteroids are effective in reducing the inflammatory aspects of DTS. Statistically significant reductions in the signs (conjunctival hyperemia) and symptoms of individuals diagnosed with keratoconjunctivitis sicca were observed in groups treated with corticosteroids (loteprednol etabonate ophthalmic suspension, 0.5%) compared with those treated with placebo (level I) [105]. Statistically significant improvements were also observed in individuals with keratoconjunctivitis sicca (associated with Sjögren syndrome) who were treated with corticosteroids (nonpreserved methylprednisolone), followed by the insertion of punctal plugs after 2 weeks, with regard to clinical signs (corneal fluorescein staining) and ocular symptoms, compared with individuals receiving punctal plug insertion alone (level I) [124]. Corticosteroid treatment of individuals with keratoconjunctivitis sicca, with and without Sjögren syndrome, resulted in significantly greater improvements in ocular symptoms and signs (fluorescein and rose bengal staining) compared with treatment with artificial tears alone or nonsteroidal anti-inflammatory drops (flurbiprofen) along with artificial tears (level I) [125].

Members of the DTS Panel have used a dosage regimen for topical corticosteroids of one to four times per day (OU) for 2–4 weeks for the treatment of tear deficiency, as appropriate, according to the severity of the condition. Although all ophthalmic corticosteroid formulations are considered suitable for the treatment of inflammation associated with tear deficiency, clinicians are advised to consider formulations that may decrease the risk of steroid-associated adverse effects (e.g., fluorometholone, loteprednol etabonate, and low-concentration [0.01%], preservative-free dexamethasone), such as elevated intraocular pressure (IOP) and cataract formation, for patients considered at risk for such a response [105,126–128].

#### Moisture chamber eyewear

Environmental factors, such as low humidity, drafts, and windy conditions, can exacerbate the symptoms of tear deficiency. Conversely, increases in periocular humidity have been reported to increase the thickness of the tear film lipid layer and lengthen the interblink time interval. The use of eyeglasses with side shields or goggles offers a noninvasive option to reduce environmental desiccating stress while increasing periocular humidity. Patients may directly purchase wrap-around glasses or specialty goggles [6,129,130].

#### Punctal plugs and cautery occlusion

Enhanced retention of tears on the ocular surface can be achieved by blocking the pathway for tear clearance and drainage. Increasing retention of the tear film on the ocular surface offers a complementary strategy to enhance aqueous production via other therapeutic options. Punctal occlusion can be achieved through the insertion of plugs or thermal cautery. Different types of punctal plugs may be used to offer temporary blockage of tear film drainage, such as with absorbable collagen and polymer plugs, or placed for an indefinite time frame through the use of nonabsorbable silicone or thermal labile polymer plugs. Permanent punctal occlusion in the form of thermal or laser cautery may be performed on patients. Before permanently occluding the puncta, a trial period with silicone punctal plugs can be beneficial to evaluate the risk of epiphora [4,131].

Multiple studies have demonstrated the clinical efficacy of punctal occlusion in the reduction of the clinical signs and symptoms of tear deficiency (level II). Improvement in the symptoms of DTS was observed in the majority of patients in these studies (74–86%). Clinical improvements related to the signs of DTS were observed, including corneal staining, TBUT, and tear film osmolarity. Adverse effects related to punctal occlusion include epiphora, foreign body sensation, ocular irritation, infection, pyogenic granuloma, and loss of the inserted plugs (internally and externally) [132–134]. Treatment for ocular surface inflammation prior to the insertion of punctal plugs is recommended [4].

#### Autologous serum

Naturally occurring biologic fluids, such as serum, offer a lubricant that mimics the composition of natural tears in many ways and may be used as a tear supplement. The use of autologous serum ameliorates the risk of antigenicity. Serum contains many of the components present in the tear film, including immunoglobulins, enzymes, and growth factors [135,136]. Data supporting the clinical efficacy of autologous serum in randomized, well controlled studies are limited; however, clinical investigation in individuals with dry eye and Sjögren syndrome (level II/level III) reported improvement in patient symptoms, TBUT, and corneal and conjunctival staining. No complications were reported following instillation of autologous serum [137].

Preparing and maintaining the sterility of preservative-free autologous serum can be a limiting challenge in the use of this therapy. If available, a 20–50% preparation, diluted in artificial tears, may be used as a therapeutic option and applied four to eight times a day (OU) [135,137]. In areas in which autologous serum is not available or desired, other biologic fluids, including preparations of allogeneic serum [138], umbilical serum [139], and platelet lysate [140], may be used in the treatment of patients with severe forms of DED.

#### Albumin

Serum albumin is a major component of human serum and is commercially available in a purified form. As an alternative to direct preparation of serum eye drops, compounded formulations of serum albumin have been investigated in the laboratory and clinic for the treatment of dry eye. Animal models of corneal erosions using 5 or 10% serum albumin indicated a positive improvement in corneal healing and rescue of corneal epithelial cells [141,142]. Clinical evaluation of a 5% serum albumin formulation was conducted via a case series (level III) in the treatment of patients with Sjögren syndrome. Significant improvements in ocular surface staining were observed in patients following treatment with 5% albumin every 4 h after 4 weeks of treatment. No adverse events were reported with serum albumin instillation [141].

#### Topical hormones

Steroid hormones are potent signaling molecules, with regulatory effects on many physiologic processes when applied systemically. Hormone replacement therapy, particularly estrogen alone, has been associated with an increased risk of dry eye in epidemiologic studies of postmenopausal women [143]. However, the effect is unclear because evidence of a benefit to patient symptoms with hormone replacement therapy has also been reported [144]. During the aging process, both men and women generally experience a decrease in the production of androgens and women experience a decrease in the production of estrogen [145]. The presence of receptors for sex hormones on the lacrimal and meibomian glands suggests a regulatory role in the function of these glands, with a potential effect on DTS [146–148].

Although data from clinical investigations into the efficacy of topical hormone therapy for the treatment of tear deficiency are currently limited, the reported evidence encourages further investigation. A case study review [149] and anecdotal reports (level III) from clinicians indicate that the use of topical hormone therapy may be beneficial in the treatment of the ocular signs and symptoms of patients with a deficient tear film. Topical application of medroxyprogesterone has been used in the treatment of alkali burns and is associated with a reduction in corneal perforation, ulceration, and healing of persistent corneal epithelial defects (level II) [150].

Patients with tear deficiency have been treated with compounded formulations of medroxyprogesterone acetate, 1%; progesterone, 0.5%/testosterone, 0.5%; and dehydroepiandrosterone (DHEA), 0.5 or 1.0%, all of which may be prepared at compounding pharmacies, with treatment instilled two to six times per day (OU). No ocular adverse events have been reported with the use of topical hormone therapy for DTS. The safety and efficacy of compounded hormone ophthalmic preparations have not been fully investigated, and investigational uses are left to the judgment of each clinician.

#### Topical dapsone

Dapsone, or diaminodiphenyl sulfone, is available for oral administration as a systemic antimicrobial agent for the treatment of skin conditions (e.g., dermatitis herpetiformis) or acne vulgaris as a topical agent (5% gel) [151,152]. Although dapsone is considered an anti-infective, anti-inflammatory/immunosuppressive properties have also been recognized. Dapsone has also been used systemically in the treatment of ocular mucous membrane pemphigoid [153].

The clinical evidence pertaining to the safety and efficacy of topical dapsone for the treatment of ocular inflammatory conditions such as DTS is limited to case studies and anecdotal reports (level III). Compounded dapsone (0.25%) has been used for the treatment of patients with tear deficiency as an innovative approach to address the ocular inflammation associated with this condition. A dosage regimen of four times a day for 2 weeks followed by twice-daily administration for an indefinite period has been reported to be effective in the treatment of tear deficiency. No ocular complications or adverse events have been reported with the use of topical dapsone in the treatment of DTS [154]. The safety and efficacy of compounded dapsone for ocular use has not been fully evaluated, and investigational use is to be determined by the judgment of each clinician.

#### Tacrolimus

Tacrolimus is a compound belonging to the macrolide class of antibiotics that also exerts anti-inflammatory immunosuppressive effects. Tacrolimus ointment is indicated for dermatologic use (e.g., atopic dermatitis). The 0.03 and 0.1% formulations are commercially available, and other concentrations can be obtained from a compounding pharmacy [155]. Similar to cyclosporine, tacrolimus functions as a calcineurin inhibitor. Anti-inflammatory and immunosuppressive activity has been reported through inhibition of lymphocyte activation and the release of inflammatory mediators, including interleukin-4, interleukin-8, and tumor necrosis factor-α [156].

Limited data regarding the direct use of topical tacrolimus for the treatment of DTS are available. The results of the use of topical tacrolimus (0.03%) for the treatment of a case series (level III) of patients with Sjögren syndrome indicate that a significant improvement in the clinical signs of tear deficiency (fluorescein and rose bengal staining as well as TBUT and Schirmer test scores) was observed following twice-daily treatment with a compounded formulation. Mild ocular discomfort was reported following instillation of the topical tacrolimus formulation [157]. The safety and efficacy of tacrolimus for ocular use has not been fully evaluated, and investigational use is to be determined by the judgment of each clinician.

#### Bandage contact lenses and scleral contact lenses

Soft contact lenses have become widely adopted for use in alleviating discomfort and improving epithelial healing associated with ocular surgical procedures, such as refractive surgery [158]. Clinical studies have investigated the use of bandage contact lenses in the treatment of dry eye and other ocular conditions associated with aqueous deficiency. Symptomatic relief and a reduction of the expression of inflammatory markers in the tear film (MMPs) were observed in association with the use of bandage contact lenses to promote corneal epithelial healing (level II) [159]. Significant improvements in best-corrected visual acuity, corneal staining, and ocular symptoms were observed in patients with severe dry eye due to Sjögren syndrome who used bandage contact lenses (level II) [160]. Bandage contact lenses are also recommended as a treatment option for the management of ocular conditions associated with aqueous deficiency, such as filamentary keratitis [161]. Potential safety concerns associated with the use of bandage contact lenses include infection, edema, ocular irritation/discomfort, and reduced visual acuity [159–161].

Scleral contact lenses are specialty devices that are fitted to rest on the conjunctiva and sclera rather than on the cornea. The lens material is generally rigid in nature and constructed to be gas permeable, with the capacity to hold a reservoir of fluid under the lens, thereby hydrating and protecting the cornea from exposure and abrasion [162,163].

Scleral contact lenses are often reserved for use by patients with moderate-to-severe tear deficiency. The lengthy process required to design a custom-fitted lens and the associated expense may deter the widespread use of scleral lenses as a treatment option for tear deficiency [164]; however, improvements in patient comfort, enhanced visual acuity, and healing of persistent corneal epithelial defects have been reported. Complications and adverse events associated with reports of clinical benefit of scleral contact lenses include corneal neovascularization, which is likely associated with the patient's initial ocular condition [164–166].

#### Oral secretagogues

Oral secretagogues are pharmaceutical agents designed to stimulate secretion by target tissues via systemic administration. Cholinergic agents, such as pilocarpine and cevimeline, are available for oral administration for the treatment of dry mouth. Pilocarpine and cevimeline activate muscarinic acetylcholine receptors in the salivary and lacrimal glands to stimulate secretion. Clinical evaluation indicates that the main benefit of oral secretagogue therapy in patients with Sjögren syndrome is relieving the symptoms of dry mouth, although improvements in ocular symptoms have been observed [167]. Improvements in blurred vision and a reduction in ocular symptoms associated with reading were also observed in patients treated with oral secretagogues. Adverse effects related to the oral administration of cholinergic agents frequently include excessive sweating and gastrointestinal upset [167–169]. Members of the DTS Panel recommend referral to a primary care physician or rheumatologist for dosing and monitoring.

#### Topical *N*-acetylcysteine

*N*-Acetylcysteine (NAC) is a derivative of l-cysteine, a naturally occurring amino acid. NAC has been widely used as a systemic therapeutic agent for systemic conditions and as a topical agent to treat filamentary keratitis and corneal thinning and melts as a result of the compound's mucolytic, antioxidant, anti-inflammatory, and anticollagenolytic properties [170,171].

Topical formulations of NAC (5%) have been investigated for the treatment of dry eye. Statistically significant improvements were observed in the symptoms reported by patients with dry eye who were treated with topical NAC four times a day for 2 weeks compared with those who were treated with only artificial tears (level II). No significant differences regarding the changes in the clinical signs of dry eye were observed in the two treatment groups. Incidents of ocular burning upon instillation were reported with administration of topical NAC [172].

#### Amniotic membrane transplantation

Transplantation of the amniotic membrane (AMT), the thin inner layer of the placenta, is a recognized therapeutic option to facilitate healing of the ocular surface during treatment of severe ocular conditions. Preserved amniotic tissue may be inserted onto the ocular surface to function as a temporary graft to aid in the healing of persistent epithelial defects, acute chemical burns, or conjunctival scarring [173,174]. Amniotic membrane transplantation may be beneficial in the treatment of complications of DTS, including keratitis, recurrent corneal erosions, recurrent pterygium, and corneal neovascularization, and of those of DTS co-conspirators [175]. Commercially available preparations of amniotic membrane are available for use as a corneal bandage and are indicated for the treatment of conditions in which the ocular surface tissue is damaged, inflamed, or scarred. Adverse events associated with AMT transplantation have been reported to include residual epithelial defects, spontaneous extrusion of the implant, eye pain, and headache [175,176]. Recent advances in tissue preservation and availability may allow for a larger-scale investigation pertaining to the clinical use of AMT for the treatment of patients with tear deficiency [4,6,177].

#### Additional surgical procedures

In addition to the procedures mentioned previously in this section, surgical techniques to correct eyelid malposition, including limited tarsorrhaphy, may be used in the treatment of patients with severe tear deficiency. The ‘Exposure-related dysfunctional tear syndrome’ subsection presents an additional discussion of these procedures.

### Blepharitis/meibomian gland dysfunction (evaporative and nonevaporative) treatment options

#### Tear supplements and lubricants

The use of tear supplements and lubricants are recommended as first-line therapy for patients with blepharitis/MGD [5,7]. Formulations of artificial tears with higher viscosity, in addition to gel and ointment options, are generally considered to have an enhanced residence time on the ocular surface. Tear supplements, emulsion drops, and sprays designed to enhance the lipid component of the tear film are also available and have been evaluated in patients with evaporative tear disease associated with posterior blepharitis/MGD. Reports indicate improvements in individual symptoms, ocular surface staining, TBUT, expressibility of the meibomian glands, and lipid layer thickness (levels I–III) [178–182].

In addition to supplementation of the lipid component of the tear film, artificial tears and lubricants are used in patients with blepharitis/MGD to reduce tear film osmolarity, rehydrate the ocular surface, and assist in the removal of debris or irritants [4,5,89–91]. As discussed in the ‘Aqueous deficiency treatment options’ section, because of concerns regarding the effects of preservatives on the ocular surface [92], the authors recommend a nonpreserved formulation for patients using artificial tear supplements in excess of four times per day [92–95].

#### Eyelid hygiene/application of heat/massage

Eyelid hygiene, in combination with applied heat and massage of the lids, is often the mainstay of treatment for the different types of blepharitis and MGD. Methods for lid cleansing, heat delivery, and manipulation of the lids can vary from in-office procedures to practices easily performed in a patient's home setting [5,7]. The nature and frequency of the specific form of therapy that is recommended is based on the subtype of blepharitis/MGD present. In general, lid hygiene is emphasized for the treatment of anterior blepharitis, whereas warm compresses and massage are recommended for posterior blepharitis/MGD [5,7,183].

Cleansing and care of the eyelid and lid margin are particularly important in patients with anterior blepharitis. Crusting, debris, scurf, and scales can be removed through a combination of lid cleansers (e.g., commercially available products or a nonirritating soap/shampoo) applied using a pad, wipe, cotton applicator, or fingertip and moist heat for several minutes. Cleansing of the eyelids and lid margin may be necessary on a daily basis or several times a week, depending on the severity of the patient's condition [7]. In cases in which a patient fails to respond to general lid hygiene procedures, antibiotics are usually necessary (as noted subsequently). Where an infestation with *Demodex* is suspected, application of diluted tea tree oil (50%) via lid scrubs to the lid margin may be beneficial in reducing the signs and symptoms of blepharitis. Tea tree oil and lid scrubs may be used alone or in conjunction with a nonirritating shampoo diluted in warm water for cleansing the lid margin [7,184]. Cleaning and debridement of the lid margin may also be conducted as an in-office procedure.

Warm compresses, with or without moisture, may be applied using simple home techniques, including hot towelettes, gel packs, or mechanical methods, to apply heat during an in-office procedure. Application of heat to the eyelids allows for expression and clearance of altered meibomian gland secretions, which are often associated with MGD. Gentle massage of the eyelids in conjunction with heat application facilitates expression of the meibomian glands. Multiple clinical studies have reported evidence of the clinical benefit for patients with MGD via improvement of the signs and symptoms associated with the condition [82,185–187]; however, there is a wide variation among studies in the techniques used and the method of application of warm compresses and lid massage. Automated, in-office procedures providing heat, with massage (thermal pulsation) or without massage, may be conducted using commercially available devices [82,185–187]. Improvements in ocular signs and symptoms were observed to persist for up to 6 months following a single in-office treatment with automated application of heat and lid massage [82,185].

Alternatives to conventional lid hygiene and warm compresses/massage include meibomian gland probing and intense pulsed light therapy. Meibomian gland intraductal probing is conducted as an in-office procedure to offer symptomatic relief using a microcannula to open meibomian gland orifices in patients with obstructive MGD (level III) [188]. Intense pulsed light therapy involves the use of commercial devices during an in-office procedure to deliver high-intensity visible light therapy. Intense pulsed light is generally used to treat dermatologic conditions. Early clinical investigations into the use of the technique for patients with MGD indicate that the procedure may be beneficial in the treatment of signs and symptoms, including lid erythema and telangiectasia, with dermatologic adverse effects reported in 13% or less of individuals (level III) [189–191].

#### Nutritional supplements

Essential fatty acids obtained through dietary consumption, such as omega-3 and omega-6 fatty acids, appear to be involved in the regulation of inflammation associated with posterior blepharitis/MGD. The relative proinflammatory nature of omega-6 fatty acids versus the anti-inflammatory properties of omega-3 fatty acids has led to investigation of the role of these essential nutrients in evaporative forms of DTS. Current treatment guideline recommendations include nutritional supplements containing omega-3 fatty acids as a treatment option for patients with blepharitis/MGD [5,7].

Improvements in the clinical signs and symptoms of DED have been reported in individuals taking daily omega-3 fatty acid supplements, as discussed in the ‘Aqueous deficiency treatment options’ section [101]. Clinical studies specifically investigating the use of omega-3 fatty acid dietary supplements in the treatment of blepharitis and MGD have also been conducted (level I/II). Significant improvements in symptom scores, TBUT, meibum expression, contrast sensitivity, and inflammation of the lid margin were observed in individuals receiving omega-3 fatty acid supplements [192–194]. Clinical investigation regarding the recommended dosage and formulation of omega-3 fatty acid supplements is ongoing.

#### Topical cyclosporine

Chronic inflammation is a component of blepharitis/MGD. As such, the use of topical cyclosporine as a treatment option for these conditions as well as for aqueous deficiency has been investigated. Several small clinical studies (level II) investigating the application of topical cyclosporine in the treatment of MGD have been conducted. Significant improvements were observed in patient-reported symptoms, TBUT, ocular surface staining, telangiectasia, and lid margin vascular injection in individuals treated with topical cyclosporine compared with those treated with only control/placebo (artificial tears) [195,196] or tobramycin/dexamethasone (0.3%/0.1%) [197]. Improvements in the clinical signs of MGD were generally apparent following treatment with topical cyclosporine for 2 months or longer. Ocular burning, discomfort, and/or drug intolerance were reported following instillation of topical cyclosporine for the treatment of blepharitis/MGD [195,196]. The safety and efficacy of the use of topical cyclosporine for the treatment of blepharitis/MGD is still being investigated, and investigational use is best determined by the judgment of each clinician.

#### Topical lifitegrast

The integrin receptor antagonist lifitegrast is an immunomodulatory agent that blocks the recruitment, activation, and release of proinflammatory mediators by lymphocytes [113–115]. Chronic inflammation and EDE are frequently associated with MGD, the most common form of DED [5,25]. Topical application of a lifitegrast ophthalmic solution (5.0%) has been evaluated for the treatment of patients with DED, and is indicated for treatment of the signs and symptoms of DED [116]. At this time, the safety and efficacy of the use of topical lifitegrast has not been evaluated for the treatment of patients with MGD; however, lifitegrast may be used in the treatment of the signs and symptoms of DED that may be present in patients with MGD. Specific effects on the meibomian glands are not known.

Topical lifitegrast should be applied twice-daily to both eyes (OU), as indicated [116]. Patient response to treatment should be assessed at follow-up visits, and therapy may be maintained indefinitely, as appropriate.

#### Topical antibiotic ointment

Infectious blepharitis, one of the subtypes of anterior blepharitis, frequently involves colonization and overgrowth of microorganisms, including *Staphylococcus aureus*, coagulase-negative staphylococcal species, such as *Staphylococcus epidermidis*, and *Propionium acnes*[33,198]. Topical commercially available ointment formulations of erythromycin, bacitracin, or other antibiotic agents are recommended for the treatment of anterior blepharitis. Topical antibiotic formulations are generally recommended for use at bedtime or twice daily for 3 weeks in the treatment of anterior blepharitis [7]. Recurrences of anterior blepharitis are common; as such, periodic treatment with topical antibiotic ointments may be needed.

The precise role of pathogenic bacteria or overgrowth of normal flora in the cause of posterior blepharitis/MGD is less clear. Bacteria may often be cultured from the lid margin of patients with posterior blepharitis/MGD, and bacterial toxins may play a role in the pathogenesis of the condition and destabilization of the tear film through saponification of the lipids expressed by the meibomian glands. Irritation and inflammation from the production of inflammatory mediators is believed to contribute to the chronic nature of posterior blepharitis/MGD [199,200].

#### Azithromycin

Azithromycin is an antibiotic agent belonging to the macrolide class of therapeutic agents. Topical azithromycin (1%) is indicated for the treatment of bacterial conjunctivitis. Azithromycin has an expanded spectrum of activity and a pharmacokinetic profile that results in high tissue concentrations and an extended elimination half-life. Anti-inflammatory properties, which are distinct from the direct antibiotic effects, have been observed for azithromycin. The anti-inflammatory properties of azithromycin include inhibition of immune cell migration and the activation of nuclear factor kappa B and expression of proinflammatory cytokines and chemokines [201,202].

Clinical studies have investigated the use of oral and topical formulations of azithromycin for the treatment of anterior and posterior blepharitis/MGD. Improvements in the signs (e.g., TBUT, conjunctival redness, and ocular surface staining) and symptoms (e.g., ocular itching, eyelid itching, and ocular dryness) of meibomitis and posterior blepharitis/MGD have been observed in individuals treated with oral azithromycin, generally using pulse therapy, over the course of 1 month (level II) [203–205]. Statistically significant improvements have also been observed in patient-reported symptoms, meibomian gland plugging, quality of meibomian gland secretions, TBUT, and ocular surface staining of individuals with mixed anterior and posterior blepharitis (level II) [206,207] as well as of those with posterior blepharitis/MGD alone (level II) [208–211] who were treated with topical azithromycin. These preliminary studies reported that topical instillation of azithromycin was generally well tolerated. Additionally, spectroscopic studies indicated improvements in the phase transition temperature and lipid order structure of the meibomian gland secretions of patients treated with topical azithromycin [210]. A twice-daily dosage regimen (OU) for 2 days followed by daily administration (for 2–4 weeks of therapy) of topical azithromycin has been generally used in the preliminary investigation of the treatment of blepharitis/MGD. A pulsed regimen of topical azithromycin may also be used as needed because of its high ocular tissue distribution and prolonged elimination half-life [211]. Gastrointestinal adverse events were reported with oral administration of azithromycin [204,205]. Ocular adverse events associated with topical administration of azithromycin include eye pain, ocular irritation, and blurred vision [207,209,211]. The clinical safety and efficacy of azithromycin for the treatment of blepharitis/MGD is still under investigation, and investigational use is best determined by the judgment of each clinician.

#### Topical steroid and steroid/antibiotic combinations

Topical corticosteroid formulations, including drops or ointments, are a potent treatment option for the inflammation associated with the various subtypes of blepharitis and MGD. Combination steroid formulations that include an antibiotic may be used in the treatment of ocular inflammatory conditions, in which an infectious component is known or suspected to be part of the cause. Steroid/antibiotic formulations containing tobramycin have been evaluated for the treatment of manifestations of blepharitis (level II). Improvements in the clinical signs and symptoms (composite score) of blepharokeratoconjunctivitis were observed in individuals treated with both loteprednol etabonate/tobramycin (0.5%/0.3%) and dexamethasone/tobramycin (0.1%/0.3%) [212]. Because of potential serious adverse effects associated with topical ophthalmic steroid use, including elevated IOP and cataractogenesis, steroid formulations for blepharitis/MGD may be best reserved for control of inflammatory flare-ups and for the treatment of acute inflammatory/infectious conditions such as blepharokeratoconjunctivitis and marginal keratitis [5,7].

The authors recommend application of steroid ointment or steroid-antibiotic combination formulations to the lid or ocular surface one to four times per day for 2–4 weeks, as appropriate, according to the severity of the condition. Clinicians are advised to consider formulations that may decrease the risk of steroid-associated adverse effects [e.g., fluorometholone, loteprednol etabonate, and low-dosage dexamethasone (0.01%)] in the treatment of DED and blepharitis/MGD [105,126–128,213].

#### Topical metronidazole

Metronidazole is an antimicrobial agent in the nitroimidazole family that may be administered orally to treat systemic conditions or topically as a gel or ointment for dermatologic treatment of rosacea [214]. Metronidazole formulations have also been investigated for the treatment of ocular manifestations of rosacea and blepharitis associated with demodicosis. Clinical investigation of the treatment of patients with ocular rosacea with topical metronidazole (compounded topical formulation of 0.75%) in conjunction with lid hygiene was more effective than lid hygiene alone at improving overall eyelid scores and ocular surface signs (level II). No adverse events or complications were reported with topical application of metronidazole [214]. Clinical evidence pertaining to the efficacy of the treatment of blepharitis associated with *Demodex* infestation via case studies and a prospective study indicates that topical metronidazole in conjunction with lid hygiene or additional agents, such as ivermectin, is efficacious in reducing the counts of mites in the lash follicles and clinical signs of blepharitis, including lid margin thickening/erythema, and telangiectasia (level II/III) [215,216].

Formulations of metronidazole gel/ointment are available commercially (1–2%) and via compounding pharmacies at concentrations ranging from 0.375 to 2%. Commercially available preparations may be specifically labeled as ‘not for ophthalmic use’. DTS Panel members have used compounded metronidazole ophthalmic formulations, ointments (0.375–0.75%), and drops (0.5%) applied to the lid margin at bedtime or more frequently according to the severity of the patient's blepharitis for 4 weeks to 6 months (or longer), as necessary. The clinical safety and efficacy of topical metronidazole has not been fully evaluated for the treatment of ocular rosacea/blepharitis, and investigational use is best determined by the judgment of each clinician.

#### Tetracycline class agents

Tetracycline belongs to a class of antimicrobial agents, including the tetracycline derivatives doxycycline and minocycline, which are bacteriostatic agents with anti-inflammatory properties. Tetracycline agents have an established history for the treatment of dermatologic conditions, including acne rosacea. Use of tetracycline class agents for the treatment of blepharitis and MGD has focused primarily on their anti-inflammatory properties, along with their additional effects on bacterial products that affect the tear film rather than direct antimicrobial effects [217–219].

Tetracycline agents suppress the production of staphylococcal lipase at concentrations below the threshold required for antimicrobial activity. Bacterial lipases produce free fatty acids and diglycerides with the potential to disrupt the tear film lipids and cause ocular irritation (level I/II) [220–222]. Additional anti-inflammatory and antiangiogenic properties have been observed in basic and clinical research, indicating that tetracyclines affect a range of inflammatory mediators, including MMPs and cytokines/chemokines (level I/II) [223,224].

Clinical investigations of the oral administration of tetracycline agents for the treatment of blepharitis/MGD have indicated a significant improvement of individual-reported symptoms and ocular inflammation/irritation [5,225]. The oral dosage of tetracyclines ranges from 20 mg/day by mouth for minocycline and doxycycline to 250 mg/day by mouth for up to four times a day for tetracycline, with no general consensus regarding the standardized dosage for the treatment of blepharitis/MGD [5]. Adverse effects that have been observed in patients receiving systemic tetracycline therapy include photosensitization, nausea, and headache [5,225,226].

In an effort to provide directed treatment for ocular conditions, potentially reducing adverse effects that occur frequently in patients undergoing systemic tetracycline therapy, topical doxycycline ophthalmic formulations may be obtained from compounding pharmacies. The authors have used a 0.025–0.1% formulation of doxycycline ophthalmic solution twice daily for the treatment of chronic forms of blepharitis/MGD. Ophthalmic ointment formulations of tetracycline compounds may also be used (at bedtime), if preferred. The safety and efficacy of the use of topical compounded tetracycline class agents for the treatment of blepharitis/MGD have not been evaluated, and investigational use is best determined by the judgment of each clinician.

#### Topical clindamycin

Clindamycin is an antimicrobial agent in the lincosamide class of antibiotics. Clindamycin is effective against bacterial organisms, such as *S. aureus*, *S. epidermidis*, and *P. acnes*, and is generally used to treat systemic infections. Clindamycin may also be used to treat dermatologic conditions, such as rosacea and acne; however, very limited clinical information is available regarding the use of topical clindamycin formulations for the treatment of ocular rosacea [227,228]. Anecdotally, members of the DTS Panel have reported improvement in the signs and symptoms of patients with ocular rosacea/posterior blepharitis following treatment with compounded topical clindamycin ointment (1%) at bedtime for durations of 6 months or longer, depending on the severity of the condition. The authors did not report any ocular adverse events or complications with the topical administration of clindamycin. The safety and efficacy of the use of topical compounded clindamycin for the treatment of ocular rosacea/posterior blepharitis has not been evaluated, and investigational use is best determined by the judgment of each clinician.

#### Topical dapsone

Dapsone is an antimicrobial agent with anti-inflammatory and immunosuppressive properties. Dapsone is available in topical formulations for the treatment of acne vulgaris [151,152]. As discussed in the ‘Aqueous deficiency treatment options’ section, available evidence supporting the use of topical dapsone for DTS is limited to case studies and anecdotal reports (level III) [154]; however, the use of topical dapsone is similar in nature to that of other antimicrobial agents with anti-inflammatory/immunosuppressive properties, such as macrolides, tetracycline agents, and metronidazole. Compounded dapsone (0.25%) has been used for the treatment of patients with blepharitis/MGD to address the ocular inflammation associated with this condition. A DTS Panel member has used a treatment regimen of four times a day for 2 weeks followed by twice-daily administration for an indefinite period [154]. The safety and efficacy of compounded dapsone for ocular use has not been evaluated, and investigational use is to be determined by the judgment of each clinician.

#### Topical dehydroepiandrosterone

Changes in hormone levels associated with aging or autoimmune disease are associated with an increased risk factor for DTS. A decrease in the production of androgens generally occurs in both men and women during the aging process [142]. Basic research indicates that androgens are involved in stimulating the process of lipidogenesis in the meibomian glands [229].

Currently, no level I or level II studies have been conducted to evaluate the efficacy of topical androgen therapy for the treatment of blepharitis/MGD. Clinical evidence from a case study review [149] and anecdotal reports (level III) from clinicians indicate that the use of topical DHEA therapy may be beneficial in the treatment of the ocular signs and symptoms of patients with posterior blepharitis/MGD. Patients with posterior blepharitis/MGD have used formulations of DHEA, 0.5 or 1.0%, which can be obtained from compounding pharmacies, with treatment instilled two to six times per day (OU). No ocular adverse events or complications have been reported with the use of topical hormone therapy for DTS. The safety and efficacy of compounded hormone ophthalmic preparations have not been investigated, and investigational uses are left to the judgment of each clinician.

#### Topical *N*-acetylcysteine

In addition to the clinical evidence presented in the ‘Aqueous deficiency treatment options’ section, the efficacy of NAC in the treatment of MGD has also been evaluated. A small clinical study was conducted to assess the efficacy of topical NAC (5%) in conjunction with lid hygiene compared with preservative-free artificial tears and lid hygiene in patients with MGD (level II). Statistically significant improvements in TBUT and Schirmer scores were observed in the NAC treatment group compared with the artificial tear group following 1 month of therapy (four times a day). Significant improvements in patient-reported symptoms were observed in both groups compared with baseline [230]. A small follow-up study was conducted in patients with MGD to compare the efficacy of topical NAC with a topical combination steroid-antibiotic [betamethasone/sulfacetamide sodium (0.1%/10%)]. Statistically significant improvements in patient signs and symptoms were observed in both treatment groups after 1 month of therapy, and topical NAC appeared to be as effective as the topical steroid-antibiotic treatment. Topical NAC is reported to be well tolerated in the treatment of MGD [231].

### Goblet cell deficiency/mucin deficiency treatment options

#### Tear supplements and lubricants

The mucin component of the tear film is distributed across the inner glycocalyx, composed of polysaccharides attached to the surface of the corneal and conjunctival epithelium and soluble mucins that provide a gel-like consistency to the aqueous component of the tear film. Goblet cells present in the conjunctival epithelium secrete the majority of soluble mucin (MUC5AC) in the tear film. Goblet cell loss, with an associated reduction in tear mucins, is a contributing factor to DTS [232–234].

Artificial tears are the first-line therapy for patients with goblet cell deficiency/mucin deficiency [6]. The use of tear supplements can provide relief of symptoms associated with DTS, lubrication of the ocular surface, and hydration of the patient's tear film. The range of tear supplement options for the treatment of ocular symptoms of DTS provides clinicians an array of choices for selecting the best fit for a particular patient's profile and needs. Patients with goblet cell loss may benefit from tear supplements with enhanced viscosity agents and a balanced electrolyte composition [232,235].

As presented in the ‘Aqueous deficiency treatment options’ section, clinical studies investigating the efficacy of various formulations of tear supplements have been conducted [92]. The authors recommend that patients with goblet cell loss and/or mucin deficiency use preservative-free formulations to avoid further damage to the conjunctival epithelium, particularly if tear supplements are used more than four times per day [92–95].

#### Topical cyclosporine

Normal composition of the tear film requires a sufficient number of mucin-producing goblet cells in the conjunctiva. A reduction in the number of goblet cells in patients with dry eye has been correlated with reduced soluble mucin levels in the tear film. The density of goblet cells in the conjunctiva may be evaluated through samples collected by impression cytology [236].

In addition to the clinical evidence obtained during conduct of the topical cyclosporine, 0.05%, phase 3 registration studies indicating a significant increase in Schirmer test scores, significant increases in the density of goblet cells in the conjunctiva of patients treated with topical cyclosporine compared with those treated with vehicle were observed (level I). Goblet cell densities increased by approximately 191% in samples from individuals treated with topical cyclosporine [44,108]. An additional clinical investigation evaluated the increase in conjunctival goblet cell density in individuals treated sequentially with artificial tears followed by topical cyclosporine versus artificial tears alone. After 6 weeks of topical cyclosporine therapy, goblet cell density increases of 4.1-fold and 3.1-fold were observed in the temporal and inferior bulbar conjunctiva, respectively, of individuals in the cyclosporine group [236]. The members of the DTS Panel recommend a dosage regimen of twice-daily application of topical cyclosporine to both eyes (OU) in the treatment of goblet cell deficiency for a period of at least 6 months. Ocular burning and stinging were the most commonly reported adverse events in the individuals treated with topical cyclosporine [108]. As described in the ‘Aqueous deficiency treatment options’ section, patients should be assessed at follow-up visits and therapy may be maintained indefinitely, as appropriate.

#### Topical lifitegrast

A reduction in goblet cell density and mucin deficiency in the tear film is associated with both the aqueous-deficient and evaporative forms of DED [40–42]. The effect of topical lifitegrast treatment on goblet cell density and mucin production has not been evaluated; however, lifitegrast ophthalmic solution (5.0%) has been evaluated for the treatment of patients with DED, and is indicated for treatment of the signs and symptoms of DED [116]. Lifitegrast may be used in the treatment of the signs and symptoms of DED that may be present in patients with goblet cell loss and/or mucin deficiency.

The administration of topical lifitegrast for the treatment of the signs and symptoms of DED associated with goblet cell deficiency and/or mucin deficiency is twice-daily application to both eyes (OU), as indicated [116]. Follow-up visits should be conducted to assess the patient response to treatment, and therapy may be maintained indefinitely, as appropriate.

#### Topical secretagogues

Topical secretagogue agents available internationally (diquafosol and rebamipide) induce mucin secretion as a component of the mechanism of action. Topical therapeutic agents have been investigated in the United States, but are currently not available [120,121]. Rebamipide has been demonstrated to increase the production of mucin from conjunctival goblet cells and corneal epithelial cells [237]. Diquafosol activates the purinergic P2Y2 receptor on conjunctival cells, and an increased release of MUC5AC from goblet cells was observed following topical application of diquafosol in canine research studies [238]. Topical secretagogue formulations (diquafosol sodium ophthalmic solution, 3%, and rebamipide ophthalmic suspension, 2%) are available internationally and indicated for the treatment of dry eye [122,123].

#### Vitamin A ointment

Vitamin A (retinoids) is involved in multiple levels of cellular physiology and differentiation. Sufficient levels of vitamin A are essential for the development and maintenance of goblet cell levels in the conjunctiva [35,239]. Deficiency of vitamin A is an established risk factor for the development of dry eye, impacting the mucin component of the tear film as well as the acinar cells of the lacrimal system [6,240].

Treatment of patients who have an established deficiency of vitamin A with high oral doses is effective in replenishing goblet cells in the conjunctiva and improving healing of the corneal injury and damage (level II) [240]. Evidence for the clinical efficacy of a commercially available topical vitamin A drop formulation in the treatment of dry eye has also been observed in a study comparing treatment of patients with dry eye with topical cyclosporine, 0.05% (twice a day) or topical retinyl palmitate, 0.05% (four times a day). Significant improvements in conjunctival goblet cell densities were observed over 3 months in samples from both treatment groups compared with individuals instilling preservative-free artificial tears alone (level I). The most common ocular adverse events reported with instillation of topical vitamin A were ocular burning and stinging [241]. Vitamin A ointment can be compounded for topical ophthalmic use (all-trans-retinoic acid, 0.01%). Members of the DTS Panel have used vitamin A ointment for the treatment of patients with goblet cell deficiency at bedtime for 2–6 months according to the severity of the condition. The safety and efficacy of compounded vitamin A ointment for ocular use has not been evaluated, and investigational use is to be determined by the judgment of each clinician.

#### Moisture chamber eyewear

Eyeglasses or goggles that provide a relative increase in periocular humidity are a noninvasive method for patients to reduce the symptoms associated with a rapid TBUT, similar to treatment for aqueous tear deficiency. Educating patients to avoid drafts and windy conditions by controlling environmental factors may help reduce the severity of patient discomfort. Patients may purchase eyeglasses with side shields or specialty goggles [6,129,130].

#### Scleral contact lenses

As with patients with aqueous deficiency, scleral contact lenses designed to rest on the conjunctiva and sclera may be used to protect and maintain hydration of the cornea [162,163]. Scleral contact lenses may be a suitable treatment option for patients with moderate-to-severe goblet cell deficiency, compensating for a rapid TBUT. Custom-fitted scleral lenses offer a potential benefit to patients with evaporative tear disease and those with irregular astigmatism or complex refractive issues. Scleral contact lenses have been demonstrated to offer improvements in patient comfort, enhanced visual acuity, and healing of persistent corneal epithelial defects [164–166].

### Exposure-related treatment options

#### Tear supplements and lubricating agents

Tear supplements and/or topical lubricant gel formulations are recommended as a first-line therapy for patients with exposure-related DTS [6]. Excessive drying of the ocular surface due to exposure may occur during the waking period or at night, depending on the underlying condition. The main tear film components (mucin, lacrimal gland secretions, and lipids) may be produced in sufficient quantity in patients with exposure keratopathy; incomplete blinking or malposition of the lids, however, may extend the duration of the interblink interval beyond the stability of the tear film. Artificial tears, tear film supplements, and lubricating gels may be used to hydrate the ocular surface and to reduce elevated levels of proinflammatory mediators that may be present as a result of extended exposure (level II/III) [4,5,88–91].

Artificial tears and tear supplements with higher viscosity and gel or ointment options may be used throughout the day, as needed, or at bedtime to hydrate and protect the ocular surface from extended exposure at night due to conditions such as nocturnal lagophthalmos. Tear supplements and lubricants may be used by patients with mild-to-severe exposure keratopathy to alleviate symptoms; however, the underlying condition should be addressed to avoid long-term damage to ocular surface tissue [1]. As previously discussed, the DTS Panel recommends that patients using artificial tears or supplements more than four times per day use a nonpreserved formulation because of potential toxicity issues with the corneal epithelium [92–95].

#### Taping of the eyelid

Patients with nocturnal lagophthalmos and other conditions leading to exposure keratopathy may benefit from taping of the eyelid on the affected eye (at bedtime). Ointment, gel, or another ocular lubricant can be applied to the ocular surface or inferior fornix, and then a pad is taped into position over the closed eye, ensuring adequate closure of the lids [242].

#### Moisture chamber eyewear

Eyeglasses, goggles, or other fabricated systems that increase the relative periocular humidity are noninvasive methods for patients to reduce the symptoms associated with exposure keratopathy. In conjunction with patient education regarding the avoidance of drafts and windy conditions, use of moisture chamber eyewear may help reduce the severity of patient discomfort. Patients may directly purchase eyeglasses with side shields or specialty goggles for daytime use. Patients with nocturnal lagophthalmos may purchase sleep masks or specialty goggles for nighttime use. Systems that increase the periocular and ambient humidity may be beneficial for patients with exposure-related DTS [6,129,130,242].

#### Scleral contact lenses

Patients with moderate-to-severe exposure-related DTS may benefit from the use of scleral contact lenses. Lenses that are designed to rest on the conjunctiva and sclera may be used to protect and maintain hydration of the cornea, providing protection to the ocular surface tissue from extended exposure due to lid malposition or another anatomic defect [162,163]. Scleral contact lenses have been demonstrated to offer enhanced patient comfort and visual acuity and healing of persistent corneal epithelial defects [164–166].

#### Surgical procedures

Surgical options should be considered for the treatment of exposure keratopathy according to the underlying cause and severity of the condition. Referral of patients with anatomic disorders or significant lid or lash malposition (i.e., entropion, ectropion, trichiasis, or distichiasis) to an oculoplastic specialist may be necessary [1,4]. Restricting the size of the interpalpebral fissure through tarsorrhaphy may also be considered for patients with severe exposure keratopathy. Marginal, limited, or complete tarsorrhaphy procedures are therapeutic options, depending on the severity of the condition and response to prior therapy [1,4,242,243].

## EMERGING THERAPIES

Investigation into the pathophysiology, improved diagnostic techniques, and treatment options for DTS is an ongoing and active field of research. In addition to the treatment options listed for each DTS subtype, novel therapeutic agents are currently being advanced through late-stage clinical development.

### Topical cyclosporine

Novel formulations of topical cyclosporine for the treatment of dry eye have been proposed and investigated in the United States and internationally. Ocular formulations that have been investigated include concentrations of 0.1% cyclosporine as well as novel vehicle formulations of topical cyclosporine [244].

### Secretagogues

Rebamipide is a quinolone compound that was initially developed for the treatment of gastric ulcers. Rebamipide induces mucus secretion and has been shown to exhibit anti-inflammatory and cytoprotective properties in addition to improving wound healing. Investigation into the ocular properties of rebamipide to induce mucus secretion and induce epithelial healing as part of the treatment of dry eye has led to the approval and marketing of the compound in Japan. Clinical investigation into the safety and efficacy of rebamipide for the treatment of dry eye in the United States is ongoing [237].

Tavilermide (MIM-D3) is a peptidomimetic, a compound designed to resemble a short chain of amino acids, that is capable of stimulating mucus secretion and is also a partial agonist of the tropomyosin receptor kinase A receptor. Tavilermide functions as a peptidomimetic of nerve growth factor, a naturally occurring protein that plays a role in the survival and differentiation of neurons [245]. Early clinical evaluation of tavilermide in individuals with dry eye indicated significant improvements in signs and symptoms compared with placebo. Clinical investigation into the safety and efficacy of tavilermide for the treatment of dry eye is ongoing [245].

### Lacrimal neurostimulation

Stimulation of the neural circuits involved in control of basal and reflex tear secretion is being investigated through the use of a device designed to stimulate the nasolacrimal reflex. Improvements in the clinical signs and symptoms of patients with mild-to-severe DED were observed following administration of low-level neurostimulation to the sensory nerves in the nasal cavity [246,247]. Clinical investigation into the safety and efficacy of lacrimal neurostimulation for the treatment of dry eye is ongoing [248].

## CASE ILLUSTRATIONS

The following are example cases of how to apply the DTS Panel approach. These case studies are not meant to imply that there is only one way to handle patients with DTS, but to demonstrate the concept of applying a directed treatment approach following determination of a differential diagnosis. Optimal treatment is determined by individual assessment of each patient and his or her needs and wants (e.g., allergies and cost); by the clinician's experiences (i.e., ‘the art of medicine’); and by the options available to each clinician.

### Case 1

A 66-year-old woman complains of having burning, gritty, and irritated eyes for 1 year, with fluctuation of vision, especially when reading and using the computer.

#### Past medical history

(1) Hypertension
(2) Seasonal allergies (no ocular involvement reported at the time of the assessment)

#### Past ocular history

(1) Contact lens wearer from age 17 to age 51
(2) LASIK (laser-assisted in situ keratomileusis) OU at age 51 for distance OU

#### Systemic medications

(1) Losartan
(2) Fexofenadine as necessary

#### Ocular medications

(1) Artificial tears OU five to six times daily

#### Social history

(1) Smoker

#### Examination

(1) Visual acuity: 20/20 OU without correction (sc); distance
(2) Schirmer test scores: 2 mm OD and 3 mm OS after 5 min without anesthesia
(3) Tear osmolarity (if available): 332 mOsm/l OD; 324 mOsm/l OS
(4) Lids: 2+ (moderate) MGD with ‘toothpaste-like secretions’; 2 mm of lagophthalmos OU
(5) Conjunctiva: moderate rose bengal staining consistent with keratoconjunctivitis sicca; no conjunctival scarring
(6) Cornea: no fluorescein staining
(7) TBUT: 7 s OD; 8 s OS

#### Diagnosis

This patient has DTS secondary to:

(1) Aqueous deficiency with a low Schirmer test score and rose bengal staining consistent with keratoconjunctivitis sicca
(2) Evaporative dry eye without conjunctival scarring, with rapid TBUT OU
(3) Blepharitis (posterior with MGD)
(4) Exposure-related DTS with lagophthalmos

No DTS co-conspirators were noted, except for seasonal allergies.

#### Treatment

(1) Topical anti-inflammatory/immunomodulatory [cyclosporine (0.05%) or lifitegrast (5.0%)] OU twice daily
(2) Topical erythromycin (0.5%) ointment OU at bedtime
  (a) Macrolides have both antibiotic and anti-inflammatory effects
  (b) Azithromycin can also be used, but in this case, the advantage of the ointment will be seen with the lagophthalmos at bedtime
(3) Warm compresses OU twice daily (beneficial for MGD)
(4) Artificial tears (preservative-free) OU every 2 h as needed
(5) Follow-up in 4–6 weeks

#### Course

Patient presents in 5 weeks stating that she is ‘50% better’. She has less burning and fluctuation of vision but still experiences symptoms of DTS.

#### Examination

(1) Visual acuity: 20/20 OU sc; distance
(2) Schirmer test scores: 5 mm OD and 5 mm OS after 5 min without anesthesia
(3) Tear osmolarity (if available): 316 mOsm/l OD; 313 mOsm/l OS
(4) Lids: 1+ (mild) MGD with fewer ‘toothpaste-like secretions’; 2 mm of lagophthalmos OU
(5) Conjunctiva: mild rose bengal staining consistent with keratoconjunctivitis sicca; OD > OS, but better
(6) Cornea: no fluorescein staining
(7) TBUT: 7 s OD; 8 s OS

#### Assessment

This patient has improved overall, with less severe MGD, improved rose bengal staining, and improved tear osmolarity, but still has signs and symptoms of DTS.

#### Treatment

(1) Continue topical cyclosporine or lifitegrast OU twice daily
(2) Consider changing erythromycin OU at bedtime to a reduced frequency of administration, such as OU at bedtime for the first 2 weeks of every month
(3) Consider placing punctal plugs OU, lower lids. Schirmer test scores are still decreased. The level of healthy tears can be increased through the use of plugs now that the ocular surface inflammation has been addressed through the use of cyclosporine or lifitegrast. If inflammation is still present, topical steroids can be included as part of the treatment regimen. Some may also choose to only continue with the current regimen because definite improvement has been noted. Topical anti-inflammatory/immunomodulatory agents may continue to allow further improvement with prolonged use, and further intervention may not be needed
(4) Follow-up in 4–6 weeks

If the patient has no symptoms of DTS and no corneal staining at the follow-up visit, continue all medications and follow-up in 6 months. If she still has symptoms and/or staining, initiate another treatment option. For example, if the Schirmer test score is still low, add upper plugs. If Schirmer test scores are better, but MGD persists, add doxycycline by mouth or change from the erythromycin ointment to a topical metronidazole ointment OU at bedtime (compounded, 0.75%). Remember, an ‘additional treatment option’ may be giving the currently prescribed therapy more time to take effect. The lagophthalmos can be managed with moisture chamber eyewear and taping the lids closed at night. If the lagophthalmos continues to contribute to DTS, a referral to an oculoplastic surgeon may be necessary because of the severity of the condition and the patient's response to treatment.

### Case 2

A 33-year-old man was referred by an internist for ocular evaluation of dry eyes after ‘pink eye’.

#### Past medical history

(1) Seasonal allergies
(2) Asthma

#### Past ocular history

(1) Viral conjunctivitis (EKC) 2 months prior with pseudomembranes
(2) Further history taking; patient ‘digs out mucus’ with his fingernails

#### Examination

(1) Visual acuity: 20/20 OD; 20/20 OS; best corrected (cc)
(2) Schirmer test score: 24 mm OD and 30 mm OS after 5 min without anesthesia
(3) Lids: normal
(4) Conjunctiva: lissamine green staining consistent with mucus fishing syndrome (inferior fornix); tarsal conjunctival scarring OU; superior mild papillary conjunctivitis secondary to possible atopic conjunctivitis (history of asthma)
(5) Cornea: clear with no fluorescein staining
(6) TBUT: 3 s OD; 4 s OS
(7) MMP-9 testing (if available) positive for dry eye in both eyes

#### Diagnosis

This patient has DTS secondary to:

(1) Evaporative dry eye with conjunctival scarring
  Presumed goblet cell loss with mucin deficiency after EKC with pseudomembranes (and possibly from atopic conjunctivitis)

The DTS co-conspirators are mucus fishing syndrome and possible atopic conjunctivitis.

#### Treatment

(1) Topical cyclosporine (0.05%) OU twice daily may help with treating (a) inflammation (MMP-9 positive); (b) DED secondary to presumed goblet cell loss, based on conjunctival scarring and lack of MGD; and (c) atopic conjunctivitis
(2) Artificial tears OU as needed (nonpreserved recommended if used more than four times per day)
(3) Instruct the patient to discontinue mucus fishing and not to wipe his eyes while they are open
(4) Consider starting a mast cell stabilizer/antihistamine and/or topical steroid depending on the severity of the allergic/atopic component
(5) Follow-up in 4–6 weeks; may need to have the patient return within 4 weeks to monitor IOP according to the patient's glaucoma risk if a topical steroid is prescribed

#### Course

Patient presents in 4 weeks stating he is ‘60% better’ with respect to symptoms of DTS.

#### Examination

(1) Visual acuity: 20/20 OD; 20/20 OS; cc, distance
(2) Lids: normal OU
(3) Conjunctiva: no lissamine green staining OU
(4) Cornea: no fluorescein staining OU
(5) TBUT: 7 s OD; 5 s OS

#### Treatment

(1) Continue topical cyclosporine (0.05%) OU twice daily
(2) Consider topical lifitegrast (5.0%) therapy (substitution or additional therapy), depending on the patient's response to treatment
(3) Add vitamin A ointment (compounded; all-trans-retinoic acid 0.01%) OU at bedtime; although the patient is better, he still has symptoms and a rapid TBUT that is presumed secondary to goblet cell loss
(4) Follow-up in 4–6 weeks
(5) Consider moist chamber goggles and environmental treatments, such as a humidifier
(6) If topical steroid drops were included in the treatment regimen at the previous visit, taper over 1–2 weeks

If the patient has no symptoms and no corneal staining at the next follow-up visit, continue all medications and follow-up in 6 months. Alternatively, consider giving the currently prescribed therapy more time to take effect.

### Case 3

A 62-year-old woman is referred by an optometrist for dry eye that has not improved with artificial tears and collagen plugs. She complains of chronic irritation and a foreign body sensation.

#### Past medical history

(1) Hyperthyroidism

#### Past ocular history

(1) Dry eye
(2) Blepharitis

#### Examination

(1) Visual acuity: 20/40 OD; 20/40 OS; sc
(2) Manifest refraction (MR): OD −0.50 −0.50 × 175 20/25; OS −0.50 −0.25 × 04 20/25
(3) Schirmer test score: 22 mm OD and 18 mm OS after 5 min without anesthesia
(4) Lids: 2+ (moderate) MGD with 2+ (moderate) erythema of the lid margins
(5) Conjunctiva: superior injection OU; rose bengal staining superiorly OU with redundant conjunctiva superiorly OU; no evidence of lagophthalmos
(6) Cornea: no fluorescein staining; no evidence of lagophthalmos
(7) TBUT: >10 s OD; >10 s OS
(8) Tear osmolarity (if available): 280 mOsm/l OD; 295 mOsm/l OS

#### Diagnosis

(1) Posterior blepharitis
(2) MGD with evidence of an abnormal tear film demonstrated by the >8 mOsm/l difference in the tear osmolarity between the eyes, despite both eyes being in the normal range

The DTS co-conspirator is SLK.

#### Treatment

(1) Topical azithromycin (1.0%) OU at bedtime (helps with MGD)
(2) Warm compresses OU twice daily
(3) Loteprednol etabonate gel (0.5%) OU twice daily for 2 weeks (helps with SLK and MGD)
(4) Follow-up in 4 weeks

#### Course

At a follow-up of 4 weeks, the patient complained of minimal improvement in DTS symptoms.

#### Examination

(1) Visual acuity: no change in visual acuity with MR
(2) Lids: improvement in blepharitis and MGD
(3) Conjunctiva: persistent superior injection and superior rose bengal staining
(4) Cornea: no corneal staining

#### Treatment

Because MGD is better and DTS is controlled, the patient should continue all medications and treatment should focus on SLK. Management of the underlying SLK should be based on the severity of the condition using the clinician's preferred treatment options. The patient's IOP should be monitored if topical steroid therapy is continued.

### Case 4

A 53-year-old woman presents for a second opinion regarding her dry eyes.

#### Past medical history

(1) Rheumatoid arthritis
(2) Secondary Sjögren syndrome

#### Past ocular history

(1) ‘Significant dry eyes’ treated by her ophthalmologist with artificial tears, collagen plugs, and topical cyclosporine (no improvement with cyclosporine after use for 3 weeks)
(2) Further history taking; patient uses preservative-free artificial tears every 1 to 2 h

#### Examination

(1) Visual acuity: 20/40 OD; 20/30 OS; cc
(2) Schirmer test scores: 2 mm OD and 1 mm OS after 5 min without anesthesia
(3) Lids: 1 to 2+ (mild to moderate) MGD with collarettes, lid debris, and erythema
(4) Conjunctiva: lissamine green staining consistent with keratoconjunctivitis sicca; no plugs in place
(5) Cornea: 2+ (moderate) central fluorescein staining with filaments OU; classic lissamine green staining OU
(6) TBUT: 5 s OD; 6 s OS
(7) Tear osmolarity (if available): 312 mOsm/l OD; 334 mOsm/l OS
(8) MMP-9 is positive OU, confirming inflammation (if available)

#### Diagnosis

This patient has DTS secondary to:

(1) Moderate aqueous deficiency, with low Schirmer test scores and lissamine green staining consistent with keratoconjuctivitis sicca
(2) Evaporative dry eye without conjunctival scarring
(3) Blepharitis (anterior and posterior) with MGD

No DTS co-conspirators are seen.

#### Treatment

(1) Topical anti-inflammatory/immunomodulatory [lifitegrast (5.0%) or cyclosporine (0.05%)] OU twice daily
  (a) Previous ‘failure’ was determined to be secondary to using topical cyclosporine for only 3 weeks (too early to determine failure)
  (b) The patient experienced a ‘25% improvement’ in symptoms and stopped because it was ‘not 100%’
(2) Topical azithromycin (1.0%) OU at bedtime (macrolides have both antibiotic and anti-inflammatory effects)
  (a) This patient has both anterior (presumed *S. aureus*) and posterior blepharitis
(3) Warm compresses OU twice daily (beneficial for the MGD)
(4) Lid cleansers
(5) Omega-3 nutritional supplements
(6) Artificial tears (nonpreserved) OU every 2 h (not as needed)
(7) Follow-up in 4–6 weeks

#### Course

Patient presents in 4 weeks stating that she is ‘40% better’ in her DTS symptoms, including improved vision.

#### Examination

(1) Visual acuity: 20/25 OD; 20/20 OS; cc, distance
(2) Schirmer test scores: 2 mm OD and 3 mm OS after 5 min without anesthesia
(3) Lids: 1+ (mild) MGD with less erythema OU; no collarettes OU
(4) Conjunctiva: 1 to 2+ (mild to moderate) lissamine green staining consistent with keratoconjunctivitis sicca OU
(5) Cornea: 1+ (mild) fluorescein staining OU
(6) TBUT: 9 s OD; 9 s OS
(7) Tear osmolarity (if available): 302 mOsm/l OD; 315 mOsm/l OS

#### Treatment

(1) Continue topical anti-inflammatory/immunomodulatory therapy [lifitegrast (5.0%) or cyclosporine (0.05%)] OU twice daily because the patient is definitely better, with improved vision, improved staining, decreased MGD, and improved TBUT, despite only a 40% subjective improvement
(2) Consider changing topical azithromycin (1.0%) OU at bedtime to a reduced frequency of administration, such as the first 2 weeks of every month
(3) Place punctal plugs OU lower lids (Schirmer test scores are still decreased)
(4) Consider starting steroid drops OU twice daily if not started during the last visit
(5) Consider autologous serum tears if filaments are still present
(6) Follow-up in 4–6 weeks; may need to have the patient return within 4 weeks to monitor IOP according to the patient's glaucoma risk if a topical steroid is prescribed

Consider NAC if filaments are still present at the next follow-up visit. If the patient has no symptoms and no corneal staining at the next follow-up visit, continue all medications and follow-up in 6 months. If she still has symptoms and/or staining, initiate another treatment option. For example, if Schirmer scores are still low, add upper plugs or topical hormones (topical DHEA or medroxyprogesterone). If Schirmer scores are better but MGD persists, add doxycycline by mouth or doxycycline drops (compounded). If topical steroid drops were included in the treatment regimen at the previous visit, taper over 1–2 weeks.

## Acknowledgements

The authors thank Kurt Brubaker and MedEdicus for writing and editorial assistance. Responsibility for the content rests with the authors.

### Author disclosures

Q.B.A., MD, has had a financial agreement or affiliation during the past year with the following commercial interests in the form of Consultant/Advisory Board: Alcon; Allergan; and Bausch & Lomb Incorporated; Honoraria from promotional, advertising or non-CME services received directly from commercial interests or their Agents (e.g., Speakers Bureaus): Alcon; Allergan; and Bausch & Lomb Incorporated; Ownership Interest: Allergan.

R.M.A., MD, has had a financial agreement or affiliation during the past year with the following commercial interests in the form of Consultant/Advisory Board: Allergan; and Bausch & Lomb Incorporated; Honoraria from promotional, advertising or non-CME services received directly from commercial interests or their Agents (e.g., Speakers Bureaus): Alcon; and Bausch & Lomb Incorporated; Ownership Interest: IDOC; and Nanophthalmos, LLC; Patent Holder: IDOC; and Nanophthalmos, LLC.

K.A.B., MD, has had a financial agreement or affiliation during the past year with the following commercial interests in the form of Royalty: eyeXpress; Consultant/Advisory Board: Alcon; Allergan; Bausch & Lomb Incorporated; eyeXpress; Omeros Corporation; Shire; Sun Pharmaceutical Industries Ltd; and TearLab Corporation; Honoraria from promotional, advertising or non-CME services received directly from commercial interests or their Agents (e.g., Speakers Bureaus): Allergan; Alcon; Shire; and TearLab Corporation; Ownership Interest: Calhoun Vision, Inc; and Rapid Pathogen Screening, Inc.

J.B., MD, has had a financial agreement or affiliation during the past year with the following commercial interests in the form of Consultant/Advisory Board: Envista; Equinox; Omega Ophthalmics; and Vitamed; Ownership Interest: Equinox; and Omega Ophthalmics.

T.S.B., MD, has had a financial agreement or affiliation during the past year with the following commercial interest in the form of Honoraria from promotional, advertising or non-CME services received directly from commercial interests or their Agents (e.g., Speakers Bureaus): Allergan and Shire.

C.B., MD, has had a financial agreement or affiliation during the past year with the following commercial interests in the form of Consultant/Advisory Board: Allergan; Bausch & Lomb Incorporated; Glaukos Corporation; LENSAR, LLC; and Omeros Corporation; Contracted Research: Allergan; Bausch & Lomb Incorporated; and Glaukos Corporation; Honoraria from promotional, advertising or non-CME services received directly from commercial interests or their Agents (e.g., Speakers Bureaus): Allergan; Bausch & Lomb Incorporated; Glaukos Corporation; LENSAR, LLC; and Omeros Corporation; Ownership Interest: Calhoun Vision, Inc; CXL Ophthalmics; Glaukos Corporation; and Rapid Pathogen Screening, Inc.

J.P.G., MD, has had a financial agreement or affiliation during the past year with the following commercial interests in the form of Consultant/Advisory Board: Shire; Honoraria from promotional, advertising or non-CME services received directly from commercial interests or their Agents (e.g., Speakers Bureaus): Allergan; and Bausch & Lomb Incorporated.

D.F.G., MD, has had a financial agreement or affiliation during the past year with the following commercial interests in the form of Consultant/Advisory Board: Abbott Laboratories Inc; Alcon; Allergan; Bausch & Lomb Incorporated; Innovision Labs, Inc; Ocular Sciences, Inc; Ocular Therapeutix Inc; and Ora, Inc; Contracted Research: Abbott Laboratories Inc; Aerie Pharmaceuticals, Inc; Alcon; Allergan; Auven Therapeutics; Bausch & Lomb Incorporated; Eleven Biotherapeutics; Kala Pharmaceuticals; Mati Therapeutics, Inc; Ocular Therapeutix Inc; and OCULUS, Inc; Royalty: ALPHAEON Corporation.

D.G., MD, has had a financial agreement or affiliation during the past year with the following commercial interests in the form of Consultant/Advisory Board: Alcon; Allergan; Bausch & Lomb Incorporated; Glaukos Corporation; Omeros Corporation; and Shire; Honoraria from promotional, advertising or non-CME services received directly from commercial interests or their Agents (e.g., Speakers Bureaus): Alcon; Allergan; Bausch & Lomb Incorporated; Glaukos Corporation; Omeros Corporation; and Shire; Ownership Interest: Allergan; and Ocular Therapeutix Inc.

R.K.G., MD, MPH, has no relevant commercial relationships to disclose.

M.A.J., MD, has had a financial agreement or affiliation during the past year with the following commercial interests in the form of Consultant/Advisory Board: Allergan; Avellino Labs; Bausch & Lomb Incorporated; Carl Zeiss Meditec, Inc; Diopsys, Inc; i-optics; LENSAR, LLC; Marco; Ocular Therapeutix, Inc; Omeros Corporation; Paragon BioTeck, Inc; Rapid Pathogen Screening, Inc; ScienceBased Health; Shire; Stemnion; TearLab Corporation; and TearScience; Ownership Interest: Ace Vision Group, Inc; ArcScan; Calhoun Vision, Inc; Paragon BioTeck, Inc; and Rapid Pathogen Screening, Inc.

J.K., MD, has had a financial agreement or affiliation during the past year with the following commercial interests in the form of Consultant/Advisory Board: Alcon; Alimera Sciences; Allergan; Bio-Tissue; CheckedUp; i-Optics; Leica; and TrueVision; Contracted Research: Bausch & Lomb Incorporated; Ocular Therapeutix Inc; and Refocus Group, Inc; Honoraria from promotional, advertising or non-CME services received directly from commercial interests or their Agents (e.g., Speakers Bureaus): Alcon; and Shire; Ownership Interest: TrueVision.

T.K., MD, has had a financial agreement or affiliation during the past year with the following commercial interests in the form of Consultant/Advisory Board: Actavis; Acucela Inc; Aerie Pharmaceuticals, Inc; Alcon; Allergan; Avellino Labs; Bausch & Lomb Incorporated; CoDa Therapeutics, Inc; Foresight Biotherapeutics; Kala Pharmaceuticals; NovaBay Pharmaceuticals, Inc; Ocular Systems, Inc; Ocular Therapeutix Inc; Oculeve Inc; Omeros Corporation; Osiris; PowerVision, Inc; Presbyopia Therapies; Shire; Stealth BioTherapeutics Inc; TearLab Corporation;TearScience; and Valeant; Ownership Interest: Ocular Therapeutix Inc; Omeros Corporation; and TearScience.

J.I.L., MD, has had a financial agreement or affiliation during the past year with the following commercial interests in the form of Consultant/Advisory Board: Allergan; Bausch & Lomb Incorporated; Shire; TearLab Corporation; and Trefoil Therapeutics, LLC; Contracted Research: Alcon; Allergan; Auven Therapeutics; Bausch & Lomb Incorporated; Eleven Biotherapeutics; Refocus Group, Inc; and Shire; Honoraria from promotional, advertising or non-CME services received directly from commercial interests or their Agents (e.g., Speakers Bureaus): Allergan; Bausch & Lomb Incorporated; and Shire; Ownership Interest: Calhoun Vision, Inc; CXL Ophthalmics; Insightful Solutions, LLC; Omega Ophthalmics; Rapid Pathogen Screening, Inc; and Trefoil Therapeutics, LLC.

P.A.M., MD, has had a financial agreement or affiliation during the past year with the following commercial interests in the form of Consultant/Advisory Board: Alcon; Allergan; Shire; TearScience; and Valeant; Contracted Research: Allergan; and Shire; Ownership Interest: Rapid Pathogen Screening, Inc.

R.P.M., MD, has had a financial agreement or affiliation during the past year with the following commercial interests in the form of Consultant/Advisory Board: Abbott Laboratories Inc; Alcon; Allergan; Bausch & Lomb Incorporated; BioD, LLC; InSite Vision Incorporated; Katena Products, Inc; Nicox; OASIS Medical; Shire; and TearScience; Contracted Research: Abbott Laboratories Inc; Alcon; Allergan; Bausch & Lomb Incorporated; BioD, LLC; InSite Vision Incorporated; Katena Products, Inc; Nicox; OASIS Medical; Shire; and TearScience; Honoraria from promotional, advertising or non-CME services received directly from commercial interests or their Agents (e.g., Speakers Bureaus): Abbott Laboratories Inc; Alcon; Allergan; Bausch & Lomb Incorporated; BioD, LLC; InSite Vision Incorporated; Katena Products, Inc; Nicox; OASIS Medical; Shire; and TearScience.

M.B.M.D., MD, has had a financial agreement or affiliation during the past year with the following commercial interests in the form of Royalty/Patent Holder: CenterVue SpA; Consultant/Advisory Board: Abbott Medical Optics; Alcon; Allergan; Altaire; Bausch & Lomb Incorporated; BelpEx; Bio-Tissue; CenterVue SpA; Focus Laboratories; Naturaceutical Delivery Corporation; OcuSoft; OCULUS, Inc; Optical Express; ORCA Surgical; Perrigo Company plc; Rapid Pathogen Screening, Inc; Shire; TearLab Corporation; TearScience; and Valeant; Honoraria from promotional, advertising or non-CME services received directly from commercial interests or their Agents (e.g., Speakers Bureaus): Abbott Laboratories Inc; Allergan; Bausch & Lomb Incorporated; Bio-Tissue; Omeros Corporation; ORCA Surgical; Shire; TearLab Corporation; and TearScience; Ownership Interest: Naturaceutical Delivery Corporation; and ORCA Surgical.

M.S.M., MD, has had a financial agreement or affiliation during the past year with the following commercial interests in the form of Consultant/Advisory Board: Allergan; Omeros Corporation; Shire; and Sun Pharmaceutical Industries Ltd; Contracted Research: Eleven Biotherapeutics; EyeGate; and Kala Pharmaceuticals; Honoraria from promotional, advertising or non-CME services received directly from commercial interests or their Agents (e.g., Speakers Bureaus): Allergan; Bausch & Lomb Incorporated; Shire; Sun Pharmaceutical Industries Ltd; and TearScience; Ownership Interest: Rapid Pathogen Screening, Inc.

R.K.R., MD, has had a financial agreement or affiliation during the past year with the following commercial interests in the form of Salary: Avedro, Inc; Consultant/Advisory Board: Abbott Laboratories Inc; Alcon; Allergan; Avedro, Inc; Bausch & Lomb Incorporated; Mimetogen Pharmaceuticals; Nicox; Rapid Pathogen Screening, Inc; Santen Pharmaceutical Co, Ltd; Shire; and Valeant; Contracted Research: Abbott Laboratories Inc; Alcon; Allergan; Avedro, Inc; Bausch & Lomb Incorporated; Mimetogen Pharmaceuticals; Nicox; Rapid Pathogen Screening, Inc; Santen Pharmaceutical Co, Ltd; Shire; and Valeant; Honoraria from promotional, advertising or non-CME services received directly from commercial interests or their Agents (e.g., Speakers Bureaus): Abbott Laboratories Inc; Alcon; Allergan; Avedro, Inc; Bausch & Lomb Incorporated; Mimetogen Pharmaceuticals; Nicox; Rapid Pathogen Screening, Inc; Santen Pharmaceutical Co, Ltd; Shire; and Valeant; Ownership Interest: Avedro, Inc; Calhoun Vision, Inc; Mimetogen Pharmaceuticals; Rapid Pathogen Screening, Inc; and TearSolutions, Inc.

T.R., MD, has had a financial agreement or affiliation during the past year with the following commercial interests in the form of Consultant/Advisory Board: Abbott Medical Optics; Glaukos Corporation; and Ocular Therapeutix, Inc; Honoraria from promotional, advertising or non-CME services received directly from commercial interests or their Agents (e.g., Speakers Bureaus): Abbott Medical Optics; Bausch & Lomb Incorporated; Glaukos Corporation; Shire; and TearScience.

S.R., MD, has had a financial agreement or affiliation during the past year with the following commercial interests in the form of Consultant/Advisory Board: Alcon; Allergan; Bausch & Lomb Incorporated; Bio-Tissue; Imprimis Pharmaceuticals, Inc; Omeros Corporation; Rapid Pathogen Screening, Inc; Shire; and Sun Pharmaceutical Industries Ltd; Honoraria from promotional, advertising or non-CME services received directly from commercial interests or their Agents (e.g., Speakers Bureaus): Bausch & Lomb Incorporated; Physician Recommended Nutriceuticals; and Shire.

N.S., MD, has had a financial agreement or affiliation during the past year with the following commercial interests in the form of Consultant/Advisory Board: Alcon; Allergan; Bausch & Lomb Incorporated; and Nicox; Honoraria from promotional, advertising or non-CME services received directly from commercial interests or their Agents (e.g., Speakers Bureaus): Allergan; Bausch & Lomb Incorporated; Nicox; and Shire.

J.D.S., MD, has had a financial agreement or affiliation during the past year with the following commercial interests in the form of Consultant/Advisory Board: Allergan; and Bausch & Lomb Incorporated; Honoraria from promotional, advertising or non-CME services received directly from commercial interests or their Agents (e.g., Speakers Bureaus): Allergan; Ownership Interest: Rapid Pathogen Screening, Inc.

K.S., MD, has had a financial agreement or affiliation during the past year with the following commercial interests in the form of Consultant/Advisory Board: Abbott Laboratories Inc; Alcon; Allergan; ALPHAEON Corporation; Bausch & Lomb Incorporated; Nidek Co, Ltd; Refocus Group, Inc; and Shire; Contracted Research: Alcon; Allergan; Nidek Co, Ltd; Presbia PLC; and Refocus Group, Inc; Honoraria from promotional, advertising or non-CME services received directly from commercial interests or their Agents (e.g., Speakers Bureaus): Alcon; Allergan; Bausch & Lomb Incorporated; Nidek Co, Ltd; Presbia PLC; Refocus Group, Inc; and Shire; Ownership Interest: ALPHAEON Corporation; and Physicians Protocol.

S.T., MD, has had a financial agreement or affiliation during the past year with the following commercial interests in the form of Receipt of Intellectual Rights/Patent Holder: Ocugenesis; Consultant/Advisory Board: Abbott Laboratories Inc; Allergan; NovaBay Pharmaceuticals, Inc; and Shire; Ownership Interest: Calhoun Vision, Inc.

W.T., MD, has had a financial agreement or affiliation during the past year with the following commercial interests in the form of Consultant/Advisory Board: Abbott Medical Optics; Alcon; Allergan; Bausch & Lomb Incorporated; and Shire; Honoraria from promotional, advertising or non-CME services received directly from commercial interests or their Agents (e.g., Speakers Bureaus): Allergan; Bausch & Lomb Incorporated; and NovaBay Pharmaceuticals, Inc; Ownership Interest: ALPHAEON Corporation.

K.A.W., MD, has had a financial agreement or affiliation during the past year with the following commercial interests in the form of Royalty/Patent Holder: Ocular Systems, Inc; Consultant/Advisory Board: Abbott Laboratories Inc; Omeros Corporation; Shire; and Sun Pharmaceuticals Industries Ltd; Honoraria from promotional, advertising or non-CME services received directly from commercial interests or their Agents (e.g., Speakers Bureaus): Abbott Laboratories Inc; Omeros Corporation; Shire; and Sun Pharmaceuticals Industries Ltd; Ownership Interest: Abbott Laboratories Inc; and Omeros Corporation.

G.O.W. IV, MD, has had a financial agreement or affiliation during the past year with the following commercial interests in the form of Consultant/Advisory Board: Abbott Laboratories Inc; ACE Vision Group, Inc; AcuFocus, Inc; Alcon; Allergan; Avedro, Inc; Bausch & Lomb Incorporated; Halma plc; Omega Ophthalmics; and Perfect Lens LLC; Royalty: AcuFocus, Inc.

R.J.W., MD, has had a financial agreement or affiliation during the past year with the following commercial interests in the form of Consultant/Advisory Board: Abbott Laboratories Inc; Alcon; Bausch & Lomb Incorporated; and Omeros Corporation; Honoraria from promotional, advertising or non-CME services received directly from commercial interests or their Agents (e.g., Speakers Bureaus): Abbott Laboratories Inc; Alcon; Allergan; Bausch & Lomb Incorporated; i-Optics; MacuLogix, Inc; Omeros Corporation; Sun Ophthalmics; STAAR Surgical; and TrueVision; Ownership Interest: Calhoun Vision, Inc; DigiHealth; PogoTec; Rapid Pathogen Screening, Inc; TrueVision; and uBeam.

W.F.W., MD, has had a financial agreement or affiliation during the past year with the following commercial interests in the form of Consultant/Advisory Board: Abbott Laboratories Inc; AcuFocus, Inc; Alcon; ArcScan, Inc; Bausch & Lomb Incorporated; Calhoun Vision, Inc; Cassini; New World Medical, Inc; Omega Ophthalmics; ReVision Optics, Inc; and Zeiss; Ownership Interest: Equinox; Imprimis Pharmaceuticals, Inc; and Rapid Pathogen Screening, Inc.

E.Y., MD, has had a financial agreement or affiliation during the past year with the following commercial interests in the form of Consultant/Advisory Board: Abbott Laboratories Inc; Alcon; Allergan; ArcScan, Inc; Bausch & Lomb Incorporated; Bio-Tissue; Eyekon E.R.D Ltd; i-Optics; Kala Pharmaceuticals Inc; Ocular Therapeutix Inc; OCuSOFT; Shire; TearLab Corporation; TearScience; and Valeant; Contracted Research: Arc-Scan, Inc; Bio-Tissue; i-Optics; and Topcon Corporation; Honoraria from promotional, advertising or non-CME services received directly from commercial interests or their Agents (e.g., Speakers Bureaus): Abbott Laboratories Inc; Alcon; Allergan; Bio-Tissue; i-Optics; Ocular Therapeutix Inc; Omeros Corporation; Rapid Pathogen Screening, Inc; and Shire; Receipt of Intellectual Rights/Patent Holder: Modernizing Medicine, Inc; Rapid Pathogen Screening, Inc; and Strathspey Crown.

**CME PEER-REVIEWER DISCLOSURE:** Robert Latkany, MD, has had a financial agreement or affiliation during the past year with the following commercial interests in the form of Consultant/Advisory Board: Shire; Contracted Research: Shire; Honoraria from promotional, advertising or non-CME services received directly from commercial interests or their Agents (e.g., Speakers Bureaus): Shire.

**EDITORIAL SUPPORT DISCLOSURES:** Kurt Brubaker has had a financial agreement or affiliation during the past year with the following commercial interests in the form of Medical Writing Services: Bausch & Lomb Incorporated; Barbara Aubel; Kimberly Corbin, CHCP; Diane McArdle, PhD; Michelle Ong; and Cynthia Tornallyay, RD, MBA, CHCP, have no relevant commercial relationships to disclose.

## CME Post Test

To obtain *AMA PRA Category 1 Credit*™ for this activity, complete the CME Post Test by writing the best answer to each question in the Answer Box located on the Activity Evaluation/Credit Request form on the following page. Alternatively, you can complete the CME Post Test online at http://tinyurl.com/DTSCME.

See detailed instructions at **To Obtain *AMA PRA Category 1 Credit***™ on page 2.

1. What are the primary subtypes of DED presented in the International DEWS report?

a) ADDE, EDE, and blepharitis/meibomian gland dysfunction
b) Blepharitis and ADDE
c) ADDE and EDE
d) DTS and lid margin disease

2. According to the DTS Panel approach, DTS can be classified as which of the following subtypes?

a) Mild, moderate, or severe DTS
b) Aqueous deficiency and/or evaporative tear disease
c) Aqueous deficiency, blepharitis/MGD, goblet cell deficiency/mucin deficiency, and/or exposure-related DTS
d) Lagophthalmos, SLK, and/or superficial punctate keratitis

3. According to the DTS Panel approach for the differential diagnosis of potential DTS, the first step should include all of the following, EXCEPT:

a) Medical and ocular history
b) Current and past usage of systemic and ocular medications
c) Timing, frequency, and severity of symptoms reported by the patient
d) Family history of glaucoma

4. All of the following are important in the assessment of a patient with potential DTS, EXCEPT:

a) Evaluation of ocular surface staining
b) Wave front analysis
c) Slit-lamp examination
d) TBUT measurement

5. During the initial portion of the slit-lamp examination of a patient with potential DTS, which aspects of the ocular surface and ocular anatomical structures should be observed in detail?

a) Angle by gonioscopy
b) Presence or absence of Bowman haze
c) Tear film meniscus, lashes for any abnormalities, anterior and posterior lid margins, nature of the meibomian gland secretions, appearance of the puncta, and any anomalies of the conjunctiva and cornea
d) Endothelial cell count

6. Which of the following factors contribute to an increase in tear film hyperosmolarity?

a) Decreased aqueous production by the lacrimal gland
b) Presence of MUC5AC
c) Decreased aqueous outflow through the trabecular meshwork
d) Excess vitamin C intake

7. Which of the following diagnostic tests is indicative of inflammation associated with DED/DTS?

a) Low tear osmolarity of 285 mOsm/L in each eye
b) Positive MMP-9 test
c) High number of eosinophils on conjunctival biopsy
d) Positive India ink stain

8. Testing for serologic biomarkers may aid in the management of DED through:

a) Earlier detection of all types of DED
b) Earlier detection and directed treatment of Sjögren syndrome
c) Confirmation of patient-reported symptoms
d) Determination if patients are genetically prone to MGD

9. Which of the following is diagnostic for MGD?

a) Visualization of the meibomian gland via meibography
b) Decreased tear film meniscus
c) Poor or partial blink
d) Schirmer score <10 mm after 5 min

10. According to available evidence, which treatment option for blepharitis/MGD is recommended by the DTS Panel approach for the treatment of anterior blepharitis, in which an infectious component is known or suspected to be part of the etiology?

a) Topical cyclosporine
b) Topical *N*-acetylcysteine
c) Nutritional supplements
d) Topical antibiotic formulations

11. Which of the following anti-inflammatory agents is indicated for the treatment of DED?

a) Cefazolin, lifitegrast
b) Cyclosporine, lifitegrast
c) Brimonidine, cyclosporine
d) Levofloxacin, cyclopentolate

12. A 58-year-old woman presents with ocular dryness and discomfort and reported fluctuations in vision. A nonanesthetized Schirmer score of 5 and 7 mm is recorded OD and OS, respectively. Upon examination, reduced tear meniscus and moderate punctate corneal staining is observed following instillation of fluorescein dye. Based on the DTS Panel approach, which of the following is the most likely diagnosis?

a) Aqueous deficiency
b) Blepharitis/MGD
c) Goblet cell deficiency/mucin deficiency
d) Exposure-related DTS

13. A 65-year-old woman is referred to your clinic with persistent complaints of ocular dryness and irritation associated with reading and driving. A rapid TBUT of 3 s is observed (OU), and upon expression of the meibomian glands, cloudy, thickened secretions are noted. Trace to moderate lid erythema and telangiectasia are noted on the upper and lower lids. Based on the DTS Panel approach, which of the following is the most likely diagnosis?

a) Aqueous deficiency
b) Blepharitis/MGD
c) Goblet cell deficiency/mucin deficiency
d) Exposure-related DTS

14. A 42-year-old man presents with complaints of ocular dryness and burning associated with using the computer and windy conditions. The patient reports a history of a chemical injury to both eyes 3 years ago and frequent use of preserved artificial tears in an effort to control his symptoms. A rapid TBUT of 3–5 s is observed OD and OS, respectively. Moderate-to-mild conjunctival scarring is noted upon examination. The patient has normal appearing lids and meibomian gland secretions. Based on the DTS Panel approach, which of the following is the most likely diagnosis?

a) Aqueous deficiency
b) Blepharitis/MGD
c) Goblet cell/mucin deficiency
d) Exposure-related DTS

15. A 72-year-old woman is referred for evaluation because of reported ocular irritation and foreign body sensation (OD). The patient reports frequent irritation in the affected eye upon waking, with the discomfort diminishing, but still present, throughout the day. Moderate-to-severe inferior corneal staining is present following administration of fluorescein. The patient also reports a history of Bell palsy, with a slight partial blink and lagophthalmos OD. Tear breakup time is >10 s OU. Based on the DTS Panel approach, which of the following is the most likely diagnosis?

a) Aqueous deficiency
b) Blepharitis/MGD
c) Goblet cell deficiency/mucin deficiency
d) Exposure-related DTS
